# Imputation-Based Meta-Analysis of Severe Malaria in Three African Populations

**DOI:** 10.1371/journal.pgen.1003509

**Published:** 2013-05-23

**Authors:** Gavin Band, Quang Si Le, Luke Jostins, Matti Pirinen, Katja Kivinen, Muminatou Jallow, Fatoumatta Sisay-Joof, Kalifa Bojang, Margaret Pinder, Giorgio Sirugo, David J. Conway, Vysaul Nyirongo, David Kachala, Malcolm Molyneux, Terrie Taylor, Carolyne Ndila, Norbert Peshu, Kevin Marsh, Thomas N. Williams, Daniel Alcock, Robert Andrews, Sarah Edkins, Emma Gray, Christina Hubbart, Anna Jeffreys, Kate Rowlands, Kathrin Schuldt, Taane G. Clark, Kerrin S. Small, Yik Ying Teo, Dominic P. Kwiatkowski, Kirk A. Rockett, Jeffrey C. Barrett, Chris C. A. Spencer, Malaria Genomic Epidemiological Network ¶

**Affiliations:** 1 Wellcome Trust Centre for Human Genetics, Oxford, United Kingdom; 2 Wellcome Trust Sanger Institute, Hinxton, Cambridge, United Kingdom; 3 Medical Research Council Unit, Fajara, The Gambia; 4 Royal Victoria Teaching Hospital, Banjul, The Gambia; 5 London School of Hygiene and Tropical Medicine, London, United Kingdom; 6 Malawi-Liverpool-Wellcome Trust Clinical Research Programme, College of Medicine, University of Malawi, Blantyre, Malawi; 7 Liverpool School of Tropical Medicine, Liverpool, United Kingdom; 8 Blantyre Malaria Project, College of Medicine, University of Malawi, Blantyre, Malawi; 9 KEMRI–Wellcome Trust Research Programme, Kilifi, Kenya; 10 Department of Molecular Medicine, Bernhard Nocht Institute for Tropical Medicine, Hamburg, Germany; 11 Department of Twin Research and Genetic Epidemiology, King's College London, London, United Kingdom; 12 Saw Swee Hock School of Public Health, National University of Singapore, Singapore, Singapore; Brigham and Women's Hospital, United States of America

## Abstract

Combining data from genome-wide association studies (GWAS) conducted at different locations, using genotype imputation and fixed-effects meta-analysis, has been a powerful approach for dissecting complex disease genetics in populations of European ancestry. Here we investigate the feasibility of applying the same approach in Africa, where genetic diversity, both within and between populations, is far more extensive. We analyse genome-wide data from approximately 5,000 individuals with severe malaria and 7,000 population controls from three different locations in Africa. Our results show that the standard approach is well powered to detect known malaria susceptibility loci when sample sizes are large, and that modern methods for association analysis can control the potential confounding effects of population structure. We show that pattern of association around the haemoglobin S allele differs substantially across populations due to differences in haplotype structure. Motivated by these observations we consider new approaches to association analysis that might prove valuable for multicentre GWAS in Africa: we relax the assumptions of SNP–based fixed effect analysis; we apply Bayesian approaches to allow for heterogeneity in the effect of an allele on risk across studies; and we introduce a region-based test to allow for heterogeneity in the location of causal alleles.

## Author Summary

Malaria kills nearly a million people every year, most of whom are young children in Africa. The risk of developing severe malaria is known to be affected by genetics, but so far only a handful of genetic risk factors for malaria have been identified. We studied over a million DNA variants in over 5,000 individuals with severe malaria from the Gambia, Malawi, and Kenya, and about 7,000 healthy individuals from the same countries. Because the populations of Africa are far more genetically diverse than those in Europe, it is necessary to use statistical models that can account for both broad differences between countries and subtler differences between ethnic groups within the same community. We identified known associations at the genes *ABO* (which affects blood type) and *HBB* (which causes sickle cell disease), and showed that the latter is heterogeneous across populations. We used these findings to guide the development of statistical tests for association that take this heterogeneity into account, by modelling differences in the strength and genomic location of effect across and within African populations.

## Introduction

Severe malaria, meaning life-threatening complications of *Plasmodium falciparum* infection, kills on the order of a million African children each year [1]. However this represents only a small proportion of the total number of infected individuals, the majority of whom recover without life-threatening complications. Understanding the genetic basis of resistance to severe malaria could provide valuable insights into molecular mechanisms of pathogenesis and protective immunity that will aid the development of treatments and vaccines. It might also identify selective pressures that have shaped human physiology and susceptibility to other common diseases, because of the historical impact of malaria as a major cause of mortality in ancestral human populations.

Genome-wide association studies (GWAS) have identified thousands of genetic variants which predispose individuals to particular disease phenotypes. However, the vast majority of these studies are of non-communicable disease in collections of individuals with European ancestry. The challenges of applying these approaches to studying disease in Africa are well documented [2]; the long ancestral history of African populations has two consequences. Firstly it has led to an overall reduction in the correlation (linkage disequilibrium) between alleles at neighbouring loci. Secondly it has given rise to differences in the combinations of alleles along chromosome (haplotypes) both between, and within, geographically defined populations.

The first of these complications is problematic because GWAS rely on the correlations between causal mutations and genotyped markers to identify susceptibility variants. From a statistical perspective, unless the causal marker is typed directly, the reduced linkage disequilibrium acts to dilute association signals [3], making it hard to distinguish real effects on disease risk from apparent effects that arise from sampling. In theory this loss of power can be overcome simply by increasing the sample size or the number of typed markers [3]. Another way in which GWAS critically rely on correlation among nearby variants is via imputation based meta-analysis, which has proven a powerful tool for combining information across collections of individuals with similar ancestry. These approaches work by first obtaining genotypes at a common set of loci and then combining the statistical evidence at each locus, across collections, often by assuming the alleles to have a consistent, or fixed, effect on susceptibility. However, the differences in haplotype structure in Africa means that the correlation between any given marker locus and the causal allele may vary from one collection to the next, so the apparent effect on risk may be heterogeneous. The utility of applying the same methodology for meta-analysis in African populations is therefore unclear.

Here we describe the first imputation based meta-analysis approach in Africa to study severe malaria susceptibility across multiple African populations. We use data from large samples of individuals collected from Kenya, Malawi and Gambia which together included 5425 individuals with severe malaria and 6891 population controls. As each of the collections was typed on a different Illumina genotyping array, we used imputation (using the program IMPUTE2 [4]) to obtain genotypes at a common set of 1.3 million SNPs. We use these data to investigate the accuracy of the imputation, assess genetic structure within and between populations, describe association patterns at loci known to influence risk of severe malaria, and investigate methods for identifying new susceptibility loci.

## Results

### Datasets and genotyping

MalariaGEN partners in Gambia, Malawi and Kenya collected blood samples from children diagnosed with severe malaria, including cerebral malaria and severe malarial anaemia. As population controls, cord blood samples were collected from the same geographic areas as the cases. Ethical approval was obtained from each ethics committees at each of the partner study sites and institutions (Table S1). DNA was extracted from the blood samples and assayed at SNPs genome-wide on Illumina arrays. There are challenges specific to collecting blood from children in Africa, particularly if they are ill with severe malaria, making it hard to obtain large quantities of DNA. We performed whole-genome amplification (WGA) on a small quantity of DNA before array-based genotyping to preserve samples. The process of WGA can lead to additional experimental noise, potentially leading to genotype calling errors. To produce a robust set of genotype calls, we used three different calling algorithms to process intensity data from the Illumina arrays, separately in each of the three cohorts. A set of consensus calls was obtained by treating as missing any genotype that was discordant among algorithms (see Methods).

As high levels of missing (or discordant) genotypes are indicative of poor genotype calling (either due to poor assay performance or sporadic genotype clustering errors) we excluded SNPs with >2.5% missing genotype calls. Using the three-way calling in this way provided a robust set of SNPs for analysis and very little additional filtering of SNPs was required (Table S3).

Prior to imputation we excluded samples with outlying levels of missing or heterozygous genotypes as well as one of each pair of duplicate samples (see Table S2). Preliminary analysis of the data revealed a subset of control samples in the Malawi cohort which showed sporadic assay failure at a small number of SNPs across the genome. This type of error is hard to identify prior to analysis, and we provide a description of our observations in Text S1 and Figure S1 in case it is helpful to readers with similar data.

### Imputation across African populations

Imputation is now a well-established strategy for exploiting densely genotyped reference panels to infer genotypes at SNPs not assayed directly in a given study. For each study collection we thinned the set of SNPs to just those that passed quality control filters and were present in the HapMap3 haplotype panel [5] made available for use with the imputation program IMPUTE2 (http://mathgen.stats.ox.ac.uk/impute/impute_v2.html). We ran IMPUTE2 separately on each collection using the entire HapMap3 reference panel to obtain genotypes at a common set of 1.3 million SNPs (see Methods).

The accuracy of the genotype imputation typically depends on the correlation between typed and untyped SNPs and the similarity of haplotypes in available reference panels to those in the study samples. In both regards imputation in Africa is more challenging than in non-African populations [6]. Our study provides an opportunity to quantify the utility of imputation in this setting, and illustrates a number of issues that are relevant to other imputation-based studies in African populations.

**Figure 1:**
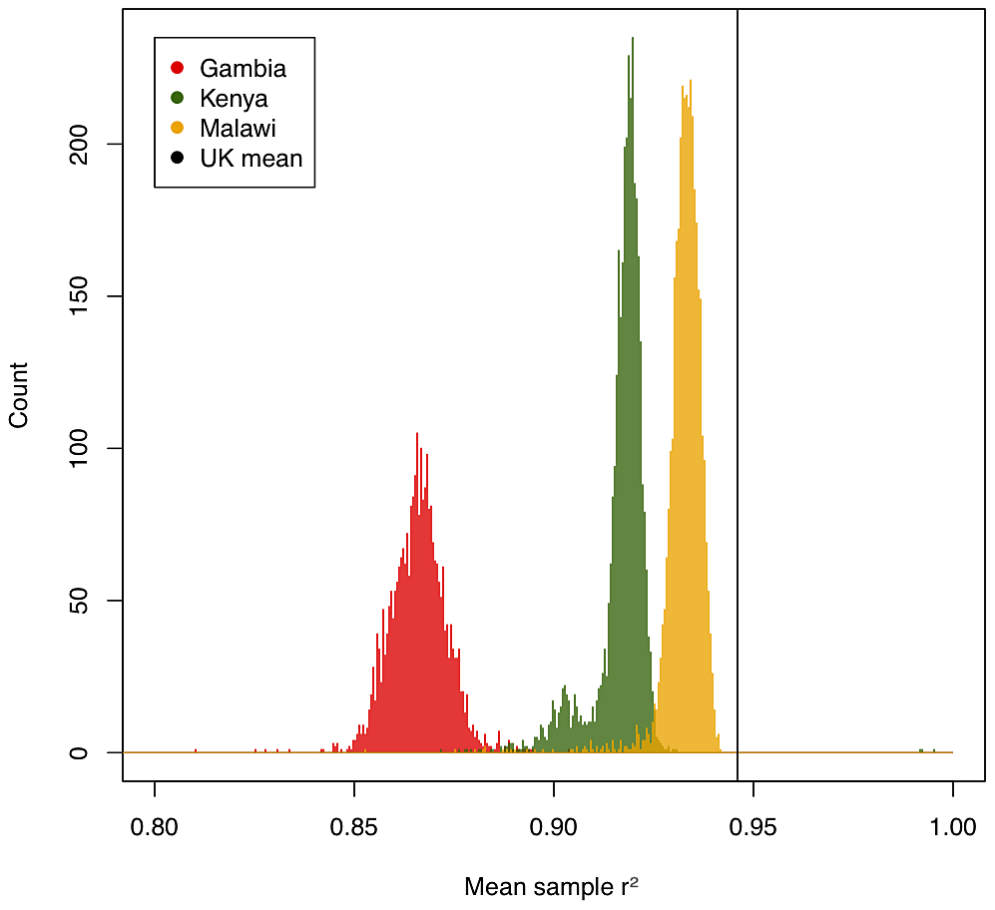
Per-sample imputation accuracy measured by r^2^ between true genotypes and genotypes predicted by imputation, averaged over imputation chunks. Black vertical line shows typical imputation accuracy in a UK population, taken from [8]. Gambian samples (red) perform worst due to the poor coverage of African variation by the Illumina 550 K platform, followed by Kenyan samples (green) on the Illumina Omni2.5M, which while dense has limited overlap with our HapMap3 reference, with Malawian samples (yellow) performing best. Note that imputation accuracy in the Kenyan sample showed a bimodal distribution driven at least partially by ethnic ancestry (Figures S12 and S13).

We assessed the accuracy of imputation by comparing the genotype calls obtained from the three-way calling described above to the probabilistic estimates for those same SNPs produced by IMPUTE2 (type 2 r^2^ in IMPUTE2). Figure 1 shows per-individual imputation accuracies broken down by country. While less accurate than typically achieved using similar genotyping arrays in European populations [7], imputation still captures the majority of common variation in these three populations (a mean type 2 r^2^ of 0.93 in Malawi, 0.92 in Kenya and 0.87 in Gambia). Common SNPs were better imputed than low-frequency SNPs, suggesting that this analysis, much like similar experiments in Europeans, will be relatively less well powered to detect associations at low frequency SNPs.

Two different types of inaccuracy are possible in imputed data: overconfidence in predicting an incorrect genotype, or underconfidence in predicting a correct genotype. We therefore evaluated the calibration of the confidence of IMPUTE2 (measured by the info score) against its actual performance at genotyped SNPs. The calibration of confidence was high across our three samples (r^2^ between predicted and true accuracy: 0.93 in Malawi, 0.92 in Kenya, 0.96 in Gambia) but, like overall accuracy, on average worse than in European samples (0.96). Quality scores were less well calibrated for low-frequency variation, but still remained relatively high across all three populations (89%, 84% and 92% respectively for variants with MAF<0.05). We included only SNPs with info score>0.75 for downstream analyses, leaving a high quality set with mean r^2^>0.9 in all samples, and less than 1% of either very overconfident (predicted r^2^>0.75, actual<0.6) or very underconfident (predicted<0.75, actual>0.9) SNPs. Taken together, these results suggest the underlying model of IMPUTE2, combined with a diverse reference panel provided by the HapMap project, is generally applicable to samples from African populations.

Despite the high performance of imputation overall, there were a number of factors that influenced relative imputation performance, including (i) genotyping platform, (ii) ethnic matching of target GWAS samples to the imputation reference panel, and (iii) homogeneity of individual GWAS collections. Our Gambian samples (typed on the Illumina 650Y array) show much poorer imputation quality (Figure 1) than our Kenyan and Malawian samples (typed on Illumina chips with >1 million SNPs). While genotyping array represents the single most important factor to imputation accuracy, two aspects of population genetics are also critical: good matching between reference and target samples (here achieved by using a cosmopolitan reference panel, likely to be improved by future reference samples of African diversity [8]) and homogeneity within a GWAS sample (illustrated by a small number of samples of differential ancestry in Kenya with poorer imputation quality, Figures S12 and S13).

### Genetic structure within and between study samples

Genetic diversity in Africa is extensive [7] and our collections derive from locations separated by thousands of miles and include individuals from several distinct ethnic groups. To characterise population structure we performed principal component analysis (PCA) across our three African collections and a set of African individuals genotyped as part of the HapMap 3 project. For this purpose we selected a set of 121029 SNPs with accurate (MAF>1%, IMPUTE2 info score>0.9) genotypes in all three study collections, and then thinned the data to reduce the correlation between neighbouring SNPs (see Methods).

To summarise the relatedness structure within our data, we similarly produced a thinned list of SNPs with good calls separately in each collection, and calculated allele sharing between all pairs of individuals at the thinned set of SNPs. The distribution of the degree of similarity between each individual and their closest relative within each study is shown in Figure S2. High levels of relatedness between individuals can violate the assumptions of standard tests of association and can dominate attempts to characterise population structure. For these reasons we iteratively removed closely related individuals and those with atypical ancestry as described in Methods. We refer to the remaining set of individuals as the “filtered set” and use them for analyses which rely on the use of principal components (PCs).

**Figure 2:**
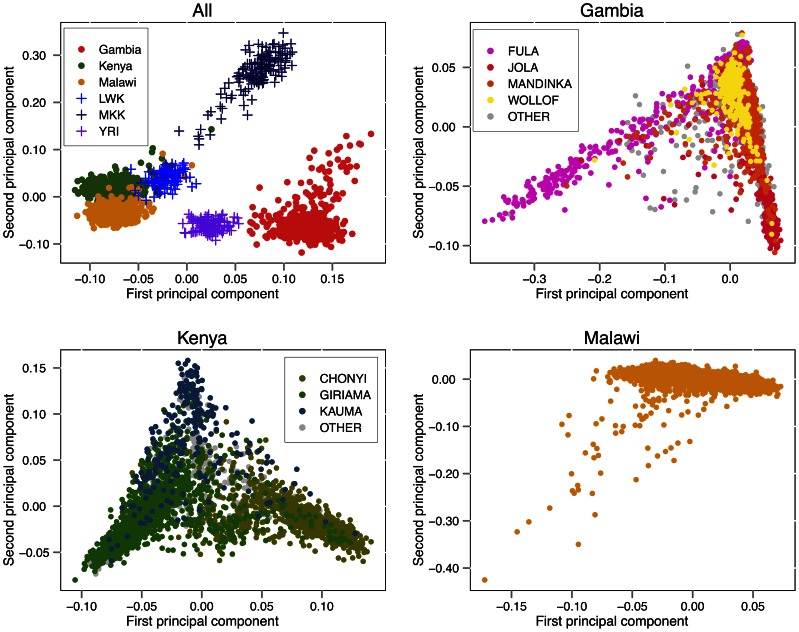
Principle components analysis. Top left: principal components analysis (PCA) of the African populations from Hapmap 3 (LWK = Luhya in Webuye, Kenya, 90 individuals; MKK = Maasai in Kinyawa, Kenya, 143 individuals; YRI = Yoruba in Ibadan, Nigeria, 113 individuals) with 500 randomly selected control samples from each of the three study cohorts. Top right, bottom left, bottom right: PCA of all non-excluded samples in each study cohort, coloured by reported ethnic group. Ethnic group is shown as “OTHER” for groups constituting less than 5% of individuals in the cohort, or where the ethnic group was unreported.

The projection of a subset of study and HapMap individuals onto the first two PCs is shown in Figure 2. Some care is needed in interpreting PCA of genetic data [9]; however, the analysis has the property that the distance between any two individuals on the plot is proportional to the genome-wide similarity in their genotypes. The relationships among our samples broadly reflect the geography and peopling of Africa; we see that East African study samples from Kenya and Malawi cluster near one another and are relatively close to HapMap Luhye individuals who are also from Kenya (LWK); and the Gambian samples cluster closer to Yoruban individuals from Nigeria (YRI), representing individuals from West Africa. The Kenyan study samples are recruited from the coastal region of Kilifi and our data confirm they are genetically distinct from the Maasai (MKK).

To characterise the genetic diversity within collections we performed PCA on each study separately and plotted all individuals on the first two principal components (Figure 2). In Gambia, the first PC separate the Fula from the rest of the sample with subsequent PC (Figure S14) stratifying further ethnic groups, as previously shown [10]. A similar relationship between genetic diversity and self-reported ancestry is seen in Kenya.

### Genome-wide meta-analysis

It is well known that subtle differences in the patterns of relatedness between case and control individuals can potentially lead to spurious signals of association [11]. We observed significant correlation between case control status and the principal components in each of studies (Table S4), and the probability that the closest relative to each sample had the same case status was significantly greater than an expected by chance (*P*<1×10^−4^ in all three cohorts).

We took two approaches to controlling for the potential bias that can result from population structure. Firstly we restricted analysis to the filtered set described above and included five PCs as covariates in a logistic regression analysis as implemented in the program SNPTEST (https://mathgen.stats.ox.ac.uk/genetics_software/snptest/snptest.html). Secondly we included all individuals passing quality control filters by modelling the covariance in case status due to relatedness between samples as a random effect in a linear model approximation, as implemented in the program MMM [12]. The latter of these approaches potentially retains more power by increasing the sample size, and provides additional robustness to population structure by modelling relatedness at all levels (or equivalently using all PCs) [13]. Empirically, the evidence for association at each SNP is similar between the two methods (Figures S3 and S4) and both reduce the overall inflation in test statistic to acceptable levels (Table S6). Throughout we assume an additive model of association, estimating a single parameter which determines the effect each copy of risk allele has on the log-odds of being a case individual, using the mixed model approach, unless otherwise stated.

To combine the evidence of association across studies we use an inverse-variance weighted fixed effects approach to calculate an estimate of the log of the odds ratio and its standard error combined across the three studies [14]. This approach has become the standard method for meta-analysis of case-control studies. We return to a discussion of the meta-analysis below.

The results of the autosomal genome-wide association analysis are shown in Figure S5. Two regions of the genome show compelling evidence of association (*P*<5×10^−8^). These include SNPs near established malaria susceptibility loci; in the beta globin (*HBB*) gene on chromosome 11 and in the ABO blood group gene (*ABO*) on chromosome 9. Several other regions show strong but not conclusive association (*P*<1×10^−6^) and are detailed in Table S7. Additional analysis using dominant, recessive or two-parameter models did not reveal any other convincing regions of association showing consistent evidence across collections, nor did direct analysis of the genotype data (Figures S9 and S10).

### Association at *HBB* and *ABO*

The strong signals of association at *HBB* and *ABO* demonstrate that, despite the additional challenges of genetic analysis in Africa, the standard approach to imputation based meta-analysis can identify loci with convincing levels of evidence when sample sizes are sufficiently large.

**Figure 3:**
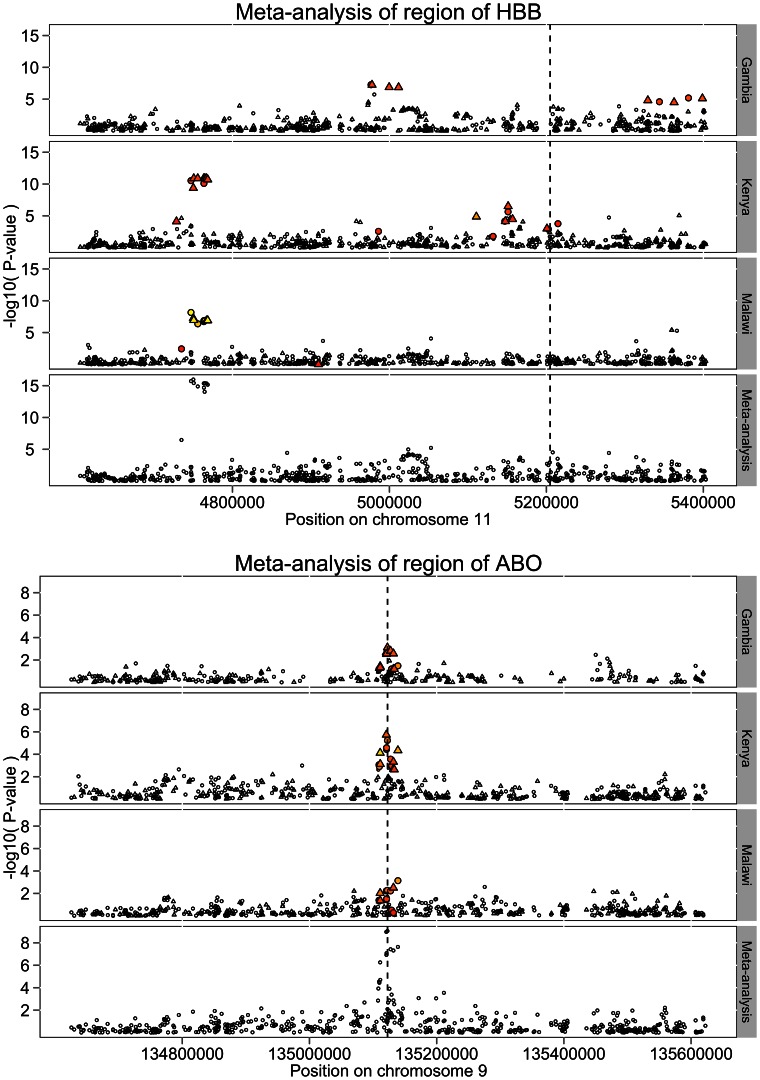
Patterns of association around the *HBB* and *ABO* loci. In each figure the top three panels of the plot is the *P* value of the logistic regression analysis using 5 PCAs in each cohort with the fixed-effect meta-analysis *P* value shown in the bottom panel. Circles represent genotyped SNP and triangles imputed SNPs. SNPs in r^2^>0.2 with the functional SNPs in each region:rs334 in *HBB* which encodes the sickle locus [HbS], and rs8176719 in *ABO* are coloured with yellow indicating more correlation and red indicating less. Vertical lines indicate the positions of the functional SNPs.

The non-synonymous variant rs334 in the *HBB* gene, whose derived allele (HbS) causes sickle-cell anaemia in homozygote individuals and is known to be strongly protective against malaria in heterozygotes, is perhaps the best known case of balancing selection in the genome [15]. The pattern of association around this region across the three cohorts is shown in Figure 3. The SNP rs334 is neither genotyped nor imputed in our genome-wide data. In its absence, the strongest signal of association is seen over 400 kb upstream of *HBB* in the Kenya and Malawi studies. The combined evidence at this locus drives the main fixed-effect meta-analysis signal. Strikingly there is very little signal of association at this position in the Gambian data, although strong evidence for association is seen closer to *HBB* gene at 200 kb upstream and, to a lesser extent, 200 kb downstream.

Direct genotyping of rs334 across the MalariaGEN data (see Methods) allows us to measure the correlation (allelic r^2^, estimated using EM algorithm) between the HbS allele and the SNPs around *HBB* in our genome-wide data. The strength of the correlation is indicated by the colour of the points in Figure 3. Association analysis of the region including genotype at rs334 as a covariate completely removes the signal (*P*>10^−4^,see Figure S6). Together these observations confirm that the heterogeneous signals of association in the three cohorts are driven by their different patterns of correlation with the causal allele rs334, probably because it arose on different haplotypes in different ancestral populations [16]. The lowest meta-analysis *P* value across the region is at rs12788102 (*P* = 1.9×10^−16^), which was imputed in Gambia, whereas at the directly typed rs334 the meta-analysis *P* = 2.8e^−36^. We note that the lowest SNPTEST meta-analysis *P* value in this region is 5.7×10^−13^ at rs17325567.

In contrast to the complex patterns of association at *HBB*, the strong meta-analysis evidence for association at *ABO* derives from a combination of signals at the same set of SNPs across the three cohorts (Figure 3). In each cohort the strength of association is moderate with *P*>1e-6, so under the assumption that malaria susceptibility loci of modest effect are rare in the genome, none of these signals are convincing in any one study. However, when combined via meta-analysis they reach established level of significance (*P*<5×10^−8^) for genome-wide analysis.

The determinants of the ABO blood group are SNPs within the *ABO* gene, and have also been typed across the MalariaGEN data, including the deletion at rs8176719 which determines AB/O groups. The correlation between rs8176719 and neighbouring SNPs is also shown in Figure 3. The pattern of association across the cohorts is similar to those typically seen in studies of European populations where the correlations between alleles at tag (marker) SNPs and the causal allele are consistent across different samples. The strongest meta-analysis signal at the locus is at rs8176722 with *P* = 8.9×10^−10^. Conditioning on rs8176719 also removes any other signals of association (*P*>10^−4^) from the region (Figure S6).

### Bayesian analysis

The differences in ancestries of the three study samples can lead to a causal SNP being differentially tagged, as observed at the *HBB* locus in our data. As a consequence the effect sizes at directly typed or imputed loci can vary between samples, even when the risk at the causal locus is the same. Moreover, variation across studies in imputation accuracy can also lead to differential levels of effect size underestimation at SNPs not genotyped directly. These effects are likely to be more important in studies across African populations and motivate approaches which relax the assumption of the same or “fixed” effect.

To investigate the impact of non-fixed effect approaches on the evidence for association we used a normal approximation to the logistic regression likelihood suggested by Wakefield [17]. One way of thinking about the approach is that it uses the study-wise estimated log-odds ratio (beta) and its standard error as summary statistics of the data (See Text S2). For each model of association we assume a prior on the log odds ratio which is normally distributed around zero with a standard deviation of 0.2 (see [18] for a discussion). By changing the prior on the covariance (or correlation) in effect sizes between studies we can formally compare models where:

1. The effects are independent across studies.
2. The effects are correlated equally between studies.
3. The effects are correlated, but to a greater degree between Malawi and Kenya (referred to as “structured effects” below).
4. The effects are the same across studies (referred to as “fixed effects” below).

(See Text S2 for details). For each model we can obtain a Bayes factor (BF) for association by comparing it with the null model where all the prior weight is on an effect size of zero. These models are similar in spirit to those employed to look at shared effects across sub-phenotypes (rather than populations) in a study of ischemic stroke [19] or at heterogeneity between studies [20].

**Figure 4:**
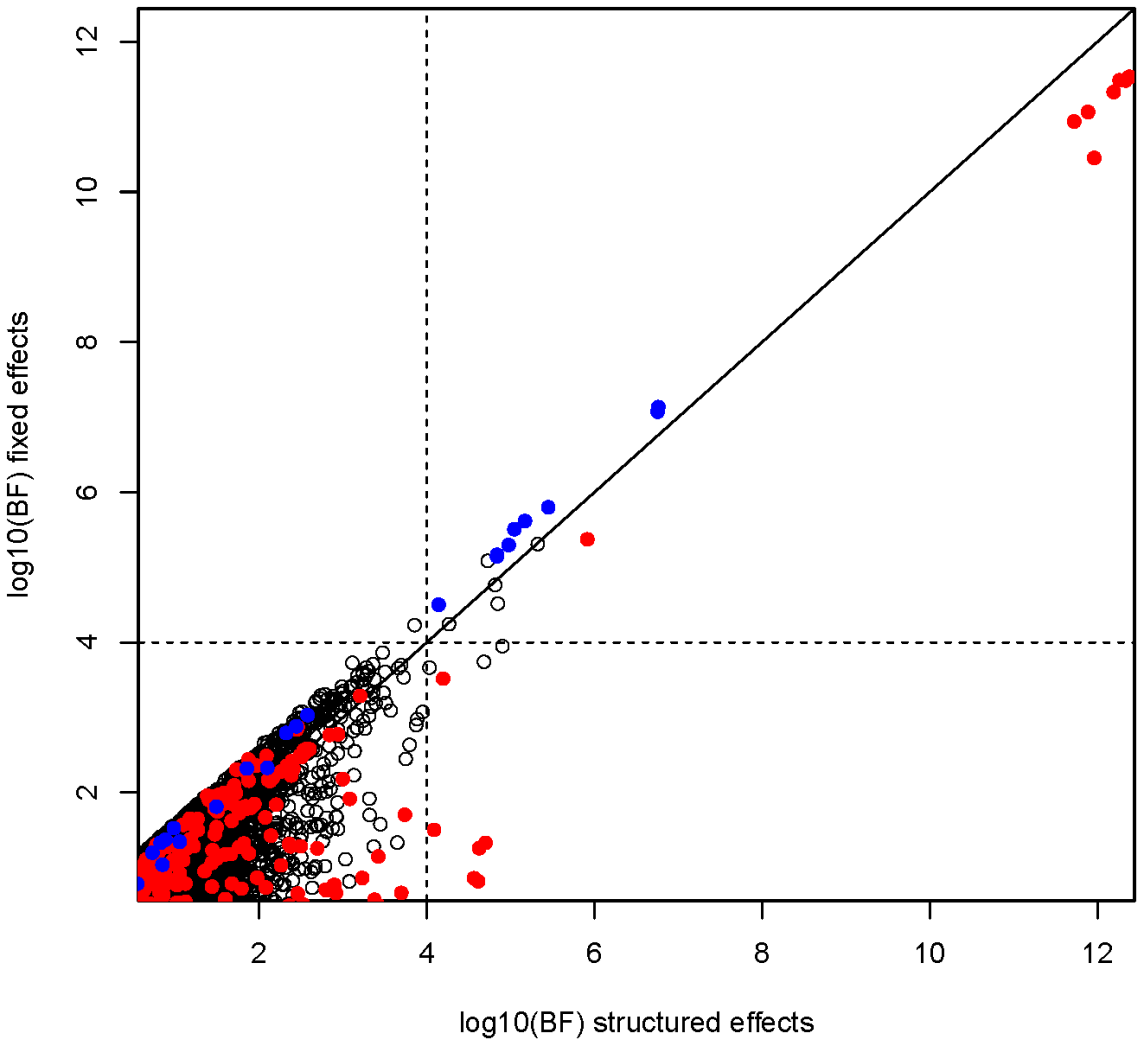
Comparison of fixed and structure effect Bayes factor at autosomal SNPs. Red dots indicate SNPs mapping to the *HBB* region in and blue dots indicate those mapping to the *ABO* region (see Figure 3). Note that points below the diagonal line have more evidence for association under a structured effects model, whereas those above the line have more evidence for association under a fixed effect model.

The genome-wide Bayes factors for the fixed effects and structured effects models are plotted against each other in Figure 4. From this plot we can see that the fixed effect BF is larger for SNPs at *ABO*, while at *HBB*, there is more evidence for association when effect sizes are allowed to vary more extensively between East and West African collections (structured effects). The posterior probability on each of the models at the SNPs in these regions is shown in Figure S17. Similar results are seen when the prior on the effect size is increased (standard deviation of 0.75; data not shown).

**Figure 5:**
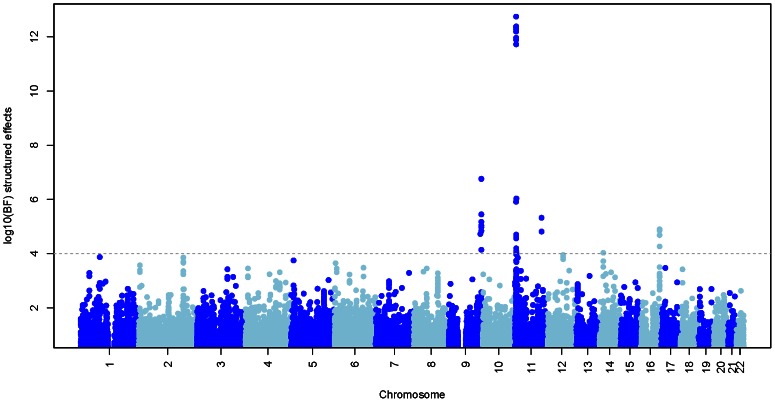
Evidence for association at approximately 1.3M autosomal SNPs. The plot shows the log10 Bayes factor comparing the structured effects model (model 4; see main text) to a model of no association. Chromosomes are coloured alternatively light and dark blue. The horizontal line indicates regions with strong evidence of association (BF>1e4).

Motivated by these observations at known malaria susceptibility loci we performed a genome-wide scan (Figure 5) to look for regions with strong evidence of association (log10(BF)>4) under model 2, 3 or 4 above. These are listed in Table S7. In large case-control samples, like those analysed here, then for common SNP (minor allele frequency >5%) the fixed effect meta-analysis *P* values are highly correlated with the fixed effect BF (see Figure S7). Nonetheless, power to detect new regions of association is highest when the prior distribution of the effect sizes across cohorts is close to the truth. We therefore advocate this approach as a way of accounting for our uncertainty in the correct meta-analysis model in terms of similarity of effect sizes between cohorts. Two regions showing over twice as much evidence for association under the structured effects model compared to the fixed effects model were on chromosome 16 in the large gene *CDH13*, where the signal of association is strongest in East Africa (Kenya and Malawi), and a region of association on chromosome 14, where the association signal is largely confined to West Africa (Gambia). The structured effects log10(BF) for these regions is 4.84 and 4.03 respectively (see Table S7).

### Regional association test

Another possible approach to identifying genetic associations across populations, where the most associated SNPs at a locus are not necessarily the same, is to base a test on all SNPs within a region [21], [22]. One way to formulate this test is to consider the causal SNP as a random effect, which is not observed, but is assumed to have a correlation structure across individuals dictated by the pattern of relatedness (or allele sharing) within the region of interest. A test for association can then be constructed by asking whether the random effect explains any of the covariance in the phenotype (case-control status), after accounting for population structure, which can be captured by including PCs as fixed effects in the model (See Text S2). As the model includes only fixed effects and a single additional random effect it can be computed using the MMM software [11]. To assess the evidence for association we used a score test statistic which has a complex, but computable, distribution under the null to calculate a *P* value. We also computed Bayes factors by specifying priors on the model parameters, in particular on the proportion of the variance explained by the region.

To exploit a region-based test we constructed allele sharing matrices (as defined in [12]) for all SNPs within 50 kb of each of approximately 20,000 genes in the genome (where there were at least 5 SNPs in each cohort). We then tested for association as described above to obtain a *P* value for each gene in each of the three study collections using the filtered data sets. To check that population structure was sufficiently accounted for by the inclusion of five PCs, we calculated the genomic inflation factor of the *P* values in each study and found them to all be less than 1.07 (after removing *HBB* and *ABO* regions). We combined evidence across cohorts using Fisher's method [23]. The meta-analysis *P* values had an acceptable genomic inflation factor of 1.071. See Figure S8. We also multiplied Bayes factors across studies to obtain a study-wide Bayes factor. We note that, unlike the fixed-effect SNP analysis, these approaches to meta-analysis do not assume that the same allele, or combination of alleles, determine susceptibility in each cohort.

As a proof of principle we applied this test to all SNPs within the *HBB* region (4.6 Mb to 5.5 Mb of chromosome 11), which covers the two peaks of association in the single SNP analysis (Figure 3). The region-based test showed evidence for association (*P*<5×10^−5^) in each cohort and had a meta-analysis *P* = 3.5×10^−17^, whereas the lowest SNPTEST meta-analysis *P* value in the region (which also uses 5 PCs as covariates) is 5.7×10^−13^. Although this region would have been discovered via either approach, this additional boost in power highlights the potential benefit of region based tests.

In the gene-based analysis the most associated region was a gene overlapping the region of strongest association at *HBB* in Malawi and Gambia (*OR51F1* from 4.73 Mb to 4.8.3 Mb, containing approximately 70 SNPs), it had a meta-analysis *P* value = 4.4×10^−11^. In contrast the gene-based signal at the *ABO* locus (*P* = 1.6×10^−5^) was significantly less than the SNP-based analysis. It is likely that the inclusion of multiple SNPs from the region and removal of assumptions about the direction of effect across cohort reduces the signal of association at this locus. However, the region-based analysis focuses attention on the approximately 20,000 annotated genes; since consensus does not yet exist on interpreting gene-based *P* values, BFs are useful in evaluating the evidence for association in the gene-based tests. For instance, if we assume that there are 20 annotated genes which contain SNPs within 50 Kb that influence malaria susceptibility then the prior odds of association are roughly 1 in 1000. In comparison, the prior probability associated with any given SNP is much lower, perhaps 1 in 100,000 [18]. Thus, a log10(BF) of 2 (BF = 100) for the region-based analysis gives the same posterior probability of association to a log10(BF) of 4 in the single-SNP analysis. Plots of the empirical distribution of the estimated proportion of the phenotypic variance explained by the regions, and a comparison of Bayesian and frequentist tests, are shown in Figure S19.

Outside the *ABO* and *HBB* regions, five regions contained genes with BF greater than 100. Although this analysis is gene focussed, it does not necessarily directly implicate specific genes but regions of the genome (see Table S7), but here we refer by gene name to the regions with the most evidence. These include the regions of the genes *BET1L* (telomere chromosome 1, log10(BF) = 2.504), *C10orf57* (chromosome 10, log10(BF) = 2.387), *MYOT* (chromosome 5, log10(BF) = 2.051)), *SMARCA5* (chromosome 4, log10(BF) = 2.04) and *ATP2B4* (chromosome 1, log10(BF) = 2.015). Interestingly, we note that the *SMARCA5* region is 250 kb upstream of the *GYPE/A/B* gene cluster which encodes the M blood group antigens, and that the variants in the *BET1L* gene have been associated with platelet volume in Europeans [24], [25]. A recent study [26] identified malaria-associated variants in *ATP2B4*.

Another benefit of a test which averages over SNPs within a region to obtain a single *P* value or BF is that it is possible to look for consistent association in collections of genes (or regions) of interest. We hypothesised that loci either previously implicated in auto-immune disease [27] (referred to below as “Immunochip regions”), associated with measurable properties of red and white blood cells and platelets [24], [28], [29], or known determinants of blood groups (obtained from the HUGO database and excluding the ABO types) might be candidates for malaria susceptibility variants. To investigate, we also calculated region-based Bayes factors for these regions and ranked them against the results from the gene-based analysis. Table S8 shows that only the Immunochip loci showed a nominally significant (*P* = 0.001) excess of high-ranking BFs (those in the top 5% of the empirical distribution) of genes. We note that, other than the *BET1L* locus, the two highest ranking (in the top 0.1% of the empirical distribution) regions include the *RUNX3* locus, implicated in ankylosing spondylitis [30] (empirical *P* = 0.0060) and the region containing the *IL12A* gene (empirical *P* = 0.0096) implicated in Celiac disease [31] and multiple sclerosis [31], [32].

### Simulations

The empirical observations described above, including the heterogeneity of signal at the *HBB* locus, and the ability of the region-based test to detect the recently identified association at the *ATP2B4* locus [26], motivates further investigation of the new approaches to association analysis. To further assess the utility of these methods we used HAPGEN [33], [34] to generate a series of simulated case-control meta-analysis datasets in ten randomly chosen genomic regions, using samples from three African populations collected as part of the HapMap project. We conducted two sets of simulation, designed to test two distinct association scenarios (see Methods). In the first set, the three populations were assigned the same underlying causal variant (with an odds ratios of 1.2 per allele), but the causal variant was assumed to be untyped. In the second set, each of the three populations had a different causal variant. We also carried out a series of null simulations (with no causal variants present at all) in order to quantify false-positive rates. We ranked all the simulations according to either the strongest evidence of association under different single SNP analyses or the region-based approach, and plotted true positive and false positive rates for all methods (Figure S18). We found that if the causal SNP is the same across populations then the fixed effect approaches, and Bayesian approaches that assume highly correlated effects, perform best. In contrast, when the causal SNP is population-specific the region-based approaches have the highest power for a fixed false positive rate. Importantly, the single SNP correlated effects Bayesian approach performs well under both scenarios, highlighting the utility of this approach when the assumption of homogeneous effect sizes across populations does not hold.

## Discussion

The purpose of our analysis was to assess the utility of imputation based meta-analysis for combining data from individuals, typed on different genotyping arrays, and sampled from different African populations, for studying Malaria susceptibility. Until recently [26], no single genome-wide analysis of malaria has revealed evidence of association strong enough to overcome the implicit low prior probability that any given SNP affects susceptibility. We show that by increasing sample size, through meta-analysis, it is possible to identify such polymorphism from diverse African populations. This reinforces the utility of applying the GWAS approach in this setting.

The two loci we identified with *P* values<5×10^−8^ have been known to influence malaria susceptibility for more than 25 years and are likely to exhibit some of the strongest effects on risk for common alleles. Extensive analysis of other phenotypes, both communicable and non-communicable, suggests that these loci are the tip of the iceberg, with many smaller effects left to be found. Identifying these regions requires additional statistical power. Primarily this can be achieved by increasing the size of the data sets by collecting more individuals or obtaining data at a denser set of SNPs, perhaps initially through imputation [6], but ultimately by whole-genome sequencing of study individuals. By typing more SNPs the difference in patterns of linkage disequilibrium between different ancestral groups, which can complicate analysis, may be mitigated as it becomes more likely that the causal variant is typed directly. Nonetheless, a combination of approaches such as the Bayesian random effect models and region-based tests outlined here may still provide additional power by relaxing the assumptions of standard SNP-by-SNP fixed effect analysis. For example, when the genetic effects are modified by the environment (such as the parasite or mosquito sub-species), or the clinical criteria for inclusion as a case individual varies between cohorts, or when different mutations arise at the same locus in different ancestral populations, even typing the causal variant may still result in effect heterogeneity. We note that the application of these new methods requires additional care because they are potentially less robust to sporadic genotyping errors in one or more cohorts. The ultimate decision about which of the approaches we have explored will be most appropriate for other researchers working on GWAS in complex populations will depend on the circumstances of individual studies. We view comparison of the models to be informative, and suggest averaging across models where a single summary of the evidence for association is preferable. Prior information on the likelihood of real, or apparent, effect heterogeneity can easily be incorporated in this approach.

Although the methods described in this paper do not confidently identify any new malaria susceptibility loci, they do highlight a set of potential candidates. For example, variation in the large gene *CDH13* has recently been conclusively associated with adiponectin levels [35] and other metabolic traits [36]. The chromosome 4 gene-based association may further implicate glycophorins A, B and E which encode the M blood group antigens and are potential receptors for the malaria parasite *P. falciparum*. The signal of association at *ATP2BA* coincides with the findings of a recent GWAS study in Ghana [26] and is of potential functional significance as it encodes the major calcium-transporting ATPase on the erythrocyte plasma membrane. It is also just upstream of *LAX1*, a transmembrane protein expressed in peripheral blood lymphocytes and implicated in T and B cell responsiveness to stimulation [37]. Further data will be required to confirm if these replicate in other collections, which specific genes are involved, and how genetic variation in the region influences severe malaria susceptibility.

There has been a long standing hypothesis that high mortality from infectious disease in ancestral populations has led to selection pressures which have had an impact on human physiology. For the first time in human genetics we are in a position to test such hypotheses. The random effects models and the region-based test described here provide a statistically principled approach to looking systematically for shared, antagonistic or pleiotrophic effects across phenotypes. The identification of new malaria susceptibility loci that will result from larger studies will empower investigations of this kind as well as providing desperately needed insights into the aetiology of malaria infection and the host's response.

## Methods

### Samples and partnerships

The studies and sample sets described in this manuscript form part of a larger ongoing project within the Malaria Genomic Epidemiology Network (www.malariagen.net). Here we describe partner projects from the MRC laboratories in Fajara, Gambia, The KEMRI-Wellcome Trust Unit in Kilif, Kenya and The Blantyre-Wellcome Trust Project in Blantyre, Malawi (Table S1). At each study site, cases of severe malaria were recruited on admission to hospital while controls (cord blood samples) for the cases were sampled from the same populations by recruiting mothers giving birth at local maternity units. This was usually done as part of a larger programme of clinical research on malaria, designed and led by local investigators. Further details can be found by visiting the MalariaGEN website (www.malariagen.net). We define a case of severe malaria as an individual admitted to a hospital or clinic with *P. falciparum* parasites in the blood film and with clinical features of severe malaria as defined by WHO criteria [38], [39].

### Ethics statement

Study sites worked with the MalariaGEN resource centre to define best practices for ethical conduct of these genetic studies in the local setting, including the development of guidelines for obtaining informed consent [40]–[42]. Further information on policies, research and the consent process may be found on the MalariaGEN website (http://www.malariagen.net/community/ethics-governance). All research was reviewed and approvals granted by local Research Boards and Ethics committees in The Gambia: The Gambia Government/MRC Unit Joint Ethics Committee (SCC1029 and SCC670/630); Kenya: Research Ethics Committee from the KEMRI-Wellcome Research Programme, Kilifi, Kenya (SCC1192); Malawi: College of Medicine Research Ethics committee, University of Malawi and the Blantyre Malaria Project with the Malawi-Liverpool-Wellcome Trust Programme, Blantyre, Malawi (P.05/06/422) and Oxford; Oxford University Tropical Research Ethics committee (OXTREC), Oxford, United Kingdom (OXTREC 020-006). This paper is published with the permission of the Director of KEMRI.

### DNA extraction and genotyping

Sample collection and DNA extraction were undertaken at partner study sites and at the MalariaGEN Resource Centre as previously described [4]. All samples submitted to the MalariaGEN Resource Centre underwent a standard set of procedures that included quantification using picogreen, genotyping of 65 polymorphisms (including HbS - rs334, and 3 gender-typing SNPs) on the Sequenom iPLEX MassArray platform and matching to baseline clinical data (e,g gender, ethnic group and case-control status) as described in [4]. Samples meeting DNA concentration and genotyping criteria with appropriate clinical data were selected for GWAS. However, due to restrictions on the total amounts of blood and DNA collected, it was necessary to first whole-genome amplify all gDNA samples by multiple-displacement-amplification as previously described [10]. Briefly gDNA was whole-genome amplified using the REPLI-g kit (Qiagen, Crawley, UK) with the modification for increased sample volumes). All final reaction DNA concentrations were measured using PicoGreen reagent (Invitrogen, Paisley, UK) and adjusted to 100 ng/ul with low TE (10 mM Tris-HCL pH 8, 0.1 mM EDTA-Na2).

Twelve percent of samples were assessed for amplicon size range using the Agilent 2100 bioanalyser (Agilent Technologies, Stockport, UK) according to manufacturer's instructions and for genotyping efficiency using the SNP set described above. Whole-genome amplified material was submitted to the Wellcome Trust Sanger Institute for genotyping on as part of the ongoing MalariaGEN consortial Project 1 (http://www.malariagen.net/node/128). Details of the 3 datasets are described in Table S1.

### Data processing

Our processing pipeline first uses the AutoConvert function in Illumina Beeline software to convert raw read data from Illumina BeadArray (idat) files into binary genotype call (gtc) files using cluster positions and normalisation information in the (egt) files. We used the gtc files to extract the calls made by the algorithm GenCall [5], to extract the raw intensities for GenoSNP [6] and the normalised intensities for the program Illuminus [7]. We wrote custom software to split the data into chunks, and modified Illuminus and GenoSNP to accept the new format. This allowed us to parallelise the genotype calling. We then included all the three sets of genotypes, along with normalised intensity data into a single vcf file (see http://www.1000genomes.org/node/101).

### Genotype calling

Three genotype calling algorithms were specifically chosen to utilise different information in the intensity data; GenoSNP which independently calls genotypes in each individual by clustering probe intensities across SNPs; Illuminus which independently calls genotypes at each SNP by clustering probe intensities across study individuals; and Gencall which uses predetermined probe intensity information to infer genotypes at each SNP in each individual.

Each of the three algorithms can make one of four calls for a given individual at a given SNP: 0,1,2 or missing. To merge the calls we took a simple consensus approach to generate a single call for each genotype. The rules were as follows:

- If less than two algorithms were confident enough to make a call (and reported the genotype as missing) we labelled the genotype as missing.
- If all algorithms that made a call agreed we kept the inferred genotype.
- If there were any discordance between the calls we labelled the genotype as missing.

The above rule is strict in the sense that only complete agreement between algorithms that made a call leads to a genotype call in the merged data. Analysis of trio data demonstrated that this retains a relatively high fraction of calls relative to anyone calling approach and had the lowest number of Mendelian errors in terms of absolute errors made and the fraction of SNPs with one or more errors (data not shown).

### Quality control

Prior to imputation we applied quality control (QC) metrics to each cohort as follows. All QC was performed on the “consensus” genotype call, as defined above. First, we aligned each dataset to the forward strand of the reference genome using the Illumina-supplied chip manifest, and restricted attention to the set of SNPs in the HapMap 3 reference panel (obtained from the IMPUTE website). We excluded samples with missingness >10% or heterozygosity outside the range 0.25–0.35. We then proceeded to filter out SNPs based on missingness (or discordance of the consensus genotype call), minor allele frequency, and HWE *P* value. We also examined differential missingness between cases and controls in each cohort, but did not apply exclusions based on this criterion. Next, we computed pair-wise concordance between samples using a thinned set of approximately 20,000 SNPs chosen to be no closer than 100 kb apart. For each pair of highly concordant samples we removed the sample with higher missingness.

### Genotype imputation

Imputation was performed with IMPUTE 2.12, using the phased release #2 of HapMap3 from the Impute website (http://mathgen.stats.ox.ac.uk/impute/). All HapMap3 haplotypes from all populations (African and non-African) were used. The genome was split up into segments which are either 5 Mb, or have 20 000 reference SNPs (whichever is smaller), with an additional 500 kb buffer on either side of the segment. We used imputation parameter settings of k = 80 and Ne = 14000. Imputation was performed in parallel for each segment, and segments were reconstructed into chromosomes once all imputations had finished. For each cohort we examined diagnostic metrics to assess imputation performance (see Figure S11).

Accuracy of imputation was measured using IMPUTE2's “type 2 r^2^”, which is the squared correlation coefficient between actual genotypes in our GWAS dataset (discrete values of 0, 1, or 2 measured by the genotyping algorithms described above) and the expected genotype, or “dosage”, predicted by IMPUTE2 (a continuous value from 0–2). As long as variants present in the GWAS are not biased towards being easier or more difficult to impute than typical variants in HapMap3, this metric is a good representation of the accuracy of our imputed genotypes at all sites [5].

### Principal component analysis and filtered data set

To investigate population structure, and for genome-wide scans performed using SNPTEST and other tools that do not directly model relatedness, we further restricted the set of individuals as follows. For each cohort we computed a matrix (denoted R) of genome-wide allele sharing, using approximately 100,000 SNPs thinned to be at least 0.01 cM apart using the HapMap combined recombination map. Using this matrix we excluded one of each pair of individuals that were closely related. Using the same matrix R we also computed the projection of samples onto the principal components (given by the eigenvectors of R). To investigate the potential for population structure to generate false signals of association we calculated the correlation between each PC and case control status using logistic regression. The resulting *P* values are shown in Table S4 and show that case control status is significantly associated with the principal components.

To ensure individuals of unusual ancestry did not dominate analyses, we iteratively excluded individuals that were extreme outliers (>10 standard deviations away from the mean) in any of the first ten PCA components, and re-computed PCs; this resulted in a small number of further exclusions (see Table S5). Projections of samples onto the principal components are depicted in Figure 2 and Figures S14, S15, S16.

### Statistical analysis

Relatedness matrix and PCA computations were performed using QCTOOL (http://www.well.ox.ac.uk/~gav/qctool) and the generateR program included with the MMM program [12] . Association tests were performed using SNPTEST v2.3.0, using maximum likelihood estimation taking into account uncertainty in imputed genotypes, and including 5 PCAs to control for population structure. Mixed model scans and region based tests were performed using MMM, with mean-centering of genotypes, imputation of missing and uncertain genotypes. For region-based tests we computed relatedness matrices using all SNPs within the region that passed QC filters (info>0.75, MAF>0.001) and used this relatedness matrix as a random effect in the MMM program. Five PCs were included to control for population structure. For Bayesian tests, we specified the prior on the *h* parameter (see Text S2) as a beta distribution with parameters 1.5 and 100; intuitively this corresponds to a belief that the regional relatedness matrix explains relatively little (around 1%) of the overall residual variance. Pairwise LD computations were performed using QCTOOL, which uses the EM algorithm to estimate the phase of individuals heterozygous at both markers. Meta-analysis was performed using fixed-effect inverse variance weighting and Fisher's method using custom software written in C and R [43]. Bayes factors for meta-analysis models were also calculated in R. For technical details on novel methods see Text S2.

### Region definitions

For the gene based analysis regions were obtained by taking the transcript start and end positions from the refGene table of the UCSC genome browser database [44]; where a gene had multiple transcripts we used the longest transcript. This resulted in 22903 gene regions, of which 21908 were on autosomes. We then added 50 kb to start and the each end of the region before applying the regional association test and only included regions with more than 5 SNPs in each of the three cohorts.

For empirical investigation of regional association test statistics we used several lists of regions. A list of blood group antigen genes was obtained from the HUGO Gene Nomenclature Committee website (http://www.genenames.org/genefamilies/blood-group) and these were extracted from the gene based analysis described above by matching gene names. To define lists of genes associated with blood cell phenotypes, we took association signals from [28] (red blood cell traits comprising Hemoglobin concentration (Hgb), hematocrit (Hct), mean corpuscular volume (MCV), mean corpuscular hemoglobin (MCH), mean corpuscular hemoglobin concentration (MCHC) and red blood cell count (RBC)) [24], (platelet traits comprising mean platelet volume (MPV) and/or platelet count (PLT)), and [29] (white blood cell traits comprising total white blood cell count (WBC), and counts of Neutrophils, Basophils, Lymphocytes and Monocytes.) For each of the three overall phenotypes (red or white blood cell, or platelet traits) we recorded the most-associated SNP across sub-phenotypes for each reported locus from the relevant study. For each such locus, we then defined a region by finding the furthest SNP upstream and downstream of the locus having r^2^>0.5 with the associated SNP, and then moving out to the nearest recombination hotspot. If the region contained no genes we further extended it by 25 kb in each direction. This procedure is the same as those implemented in GRAIL [45]. To define a list of regions associated with auto-immune disorders we used fine-mapping regions from the Immunochip platform [27].

### Simulations of effect heterogeneity

Ten 100 Kb regions were chosen uniformly from across the autosomal genome. For each region we used the program HAPGEN (v2.1.2, with default settings used [34]) to simulate a total of 1,000 meta-analysis datasets, each consisting of 1000 cases and 1000 controls from each of the three African populations (YRI, LWK and MKK) from HapMap3 (release 2). We repeated this simulation under three scenarios of association for a total of 30,000 datasets. The scenarios considered were:

1. (Null simulations): all variants were simulated under the null model of no association (and thus cases and controls were drawn from the same distribution.)
2. (Single-variant simulations): A single causal variant was picked in the region and the three populations were simulated assuming this causal variant.
3. (Three-variant simulations): A different causal variant was chosen for each population.

Causal variants were picked at random from among those with combined MAF>0.05, and assumed to act additively on the log odds scale, with odds ratio of 1.2.

For each dataset we tested each SNP in the region for association, separately in each population, using SNPTEST. We combined effect size estimates and standard errors across populations using frequentist fixed-effect and Bayesian meta-analyses (Methods and Text S2). For Bayesian meta-analysis, prior variance on effect sizes was set to 0.2^2^ and we used between-population prior correlations of 1 (fixed effect), 0.9, 0.5, and 0 (independent effect). For the single causal variant scenario, the chosen causal variant was masked from association testing.

For each dataset and population we also computed a *P*value and Bayes factor for the regional association test (Methods and Text S2). For the single causal variant scenario, the causal variant was masked from computation of the covariance matrix. We combined *P* values using Fisher meta-analysis and multiplied Bayes factors across populations to produce a single *P* value and a single Bayes factor for the dataset.

For each of the two scenarios of association and each method of detecting association across the region (regional test with Fisher meta-analysis, regional test with Bayesian meta-analysis, best single-SNP frequentist meta-analysis, best single-SNP Bayes factor for each of the four choices of correlation parameter) we produced ROC curves (Figure S18) by combining all datasets simulated under that scenario and the null scenario, ranking by the chosen *P* value or Bayes factor, and computing empirical true and false positive rates.

### Data access

For information on access to project data see www.malariagen.net.

## Supporting Information

Figure S1 Example of cluster plot from Malawi cohort with outlying sets of individuals.
(TIF)

Figure S2 Distribution of relatedness between most-related pairs.
(TIF)

Figure S3 Comparison of logistic regression (SNPTEST) and mixed model (MMM) *P* values.
(TIF)

Figure S4 SNPs showing highly divergent *P* values between logistic regression and mixed model scans.
(TIF)

Figure S5 –log10(*P* values) for test of association using the mixed model.
(TIF)

Figure S6 Top: signal of association in the HBB region after conditioning on the genotype at the known causal locus rs334. Bottom: signal of association in the ABO region after conditioning on the genotype at rs8176719.
(TIF)

Figure S7 Comparison of meta-analysis *P* values versus Bayes factors under the fixed-effect model.
(TIF)

Figure S8 Quantile-quantile plots of the region-based test in the three cohort and in the meta-analysis. The genomic control inflation factor is given in the title of the plots.
(TIF)

Figure S9 Manhattan plot showing –log10 *P* values (thresholded at 10) for additive, dominant, heterozygote, recessive, and general models, and additive model conditional on the genotype at the sickle locus rs334, across all imputed SNPs. Meta-analysis *P* values for all three cohorts and for the East African cohorts are also shown for additive, dominant, recessive and heterozygote scans.
(TIF)

Figure S10 Manhattan plot showing –log10 *P* values (thresholded at 10) for additive, dominant, heterozygote, recessive, and general models, and additive model conditional on the genotype at the sickle locus rs334, across all non-excluded genotyped SNPs. Meta-analysis *P* values for all three cohorts and for the East African cohorts are also shown for additive, dominant, recessive and heterozygote scans.
(TIF)

Figure S11 Example output from the imputation quality control pipeline for the Kenya imputation. a) per-SNP certainty (mean maximum posterior genotype call); b) per-SNP accuracy (type2 r^2^); c) per-individual type2 r^2^, averaged across segments; d) per-segment heterozygous call accuracy (proportion of true heterozygous calls that are correctly imputed with high certainty); e) average per-SNP type2 r^2^, computed per segment and plotted against the position of the segment.
(TIF)

Figure S12 The distribution of imputation quality (measured by type2 r2) across imputed Kenyan samples. The red line is at r^2^ = 0.909, and is the minimum between the two peaks.
(TIF)

Figure S13 The distribution of ethnic groups in Kenyan samples that were imputed with higher or lower quality (as defined by the red line in Figure S12). The difference in the two distributions is highly significant (Fisher's exact test, *P* = 4×10^−4^), suggesting that ethnic differences contribute to the bimodal distribution of imputation quality seen in Figure S12.
(TIF)

Figure S14 Population-specific PCA analysis of Gambian samples.
(TIF)

Figure S15 Population-specific PCA analysis of Kenyan samples.
(TIF)

Figure S16 Population-specific PCA analysis of Malawian samples.
(TIF)

Figure S17 Comparison of fixed, structured, correlated and independent-effect models at the ABO and HBB loci. The height of each bar represents the posterior probability that the corresponding model is true, under the assumption that one of the models is true.
(TIF)

Figure S18 ROC curve showing empirical true positive rate (y-axis) against false positive rate (x-axis) for each method used to detect regional association (regional test with Fisher meta-analysis, regional test with Bayesian meta-analysis, best single-SNP frequentist meta-analysis in region, best single-SNP Bayes factor for each of the four choices of correlation parameter) under the single- variant association scenario (left) and the three-variant association scenario (right).
(TIF)

Figure S19 a) Empirical distribution, across approximately 20,000 gene regions, of the maximum likelihood estimate of the eta parameter (see Text S2), for the region-based test. Overlaid (red line) is the assumed prior distribution under the alternative used to calculate Bayes factors in the region-based analysis. b) Scatter plot of the log10 combined Bayes Factor and the -log10(Fisher's *P* value). Dotted horizontal and vertical line indicates log10(Bayes Factor) = 2 and a -log10(*P* value) = 0.0005.
(TIF)

Table S1 Details on the 3 study sites and genotyping platforms.
(DOCX)

Table S2 Pre-imputation individual QC.
(DOCX)

Table S3 Pre-imputation SNP QC.
(DOCX)

Table S4 *P* values for correlation between the first 5 PCs and case/control status.
(DOCX)

Table S5 Post-imputation sample exclusions.
(DOCX)

Table S6 Genomic Inflation factors (λ) for logistic regression and mixed-model scans.
(DOCX)

Table S7 Regions showing most association in single-SNP and regional association test analyses.
(XLSX)

Table S8 Enrichment of low region based test *P* values in three previously defined sets of regions. Each *P* value in the table results from a one-sided binomial test for an enrichment in the number of regions with empirical *P* value below the given threshold. The bottom row gives a summary of the distribution of the number of SNPs in each region. Note that the Immunochip regions contain on average more SNPs than the gene-based analysis (median = 66 (quartiles = 52, 92)) from which the empirical *P* value is calculated.
(DOCX)

Text S1 Details of quality control.
(DOCX)

Text S2 Supplementary statistical details.
(PDF)
