# Correction: Imputation-Based Meta-Analysis of Severe Malaria in Three African Populations

**DOI:** 10.1371/annotation/adc2beaf-4bee-4e22-925b-6788d62fe029

**Published:** 2013-06-27

**Authors:** Gavin Band, Quang Si Le, Luke Jostins, Matti Pirinen, Katja Kivinen, Muminatou Jallow, Fatoumatta Sisay-Joof, Kalifa Bojang, Margaret Pinder, Giorgio Sirugo, David J. Conway, Vysaul Nyirongo, David Kachala, Malcolm Molyneux, Terrie Taylor, Carolyne Ndila, Norbert Peshu, Kevin Marsh, Thomas N. Williams, Daniel Alcock, Robert Andrews, Sarah Edkins, Emma Gray, Christina Hubbart, Anna Jeffreys, Kate Rowlands, Kathrin Schuldt, Taane G. Clark, Kerrin S. Small, Yik Ying Teo, Dominic P. Kwiatkowski, Kirk A. Rockett, Jeffrey C. Barrett, Chris C. A. Spencer, Malaria Genomic Epidemiology Network

The last author's name was spelled incorrectly. The correct name is: Malaria Genomic Epidemiology Network

