# Supplementary figures and images for: Imputation-Based Meta-Analysis of Severe Malaria in Three African Populations
Source: PLoS Genet. 2013 May 23;9(5):e1003509. doi: 10.1371/journal.pgen.1003509 (PMC3662650; doi:10.1371/journal.pgen.1003509)

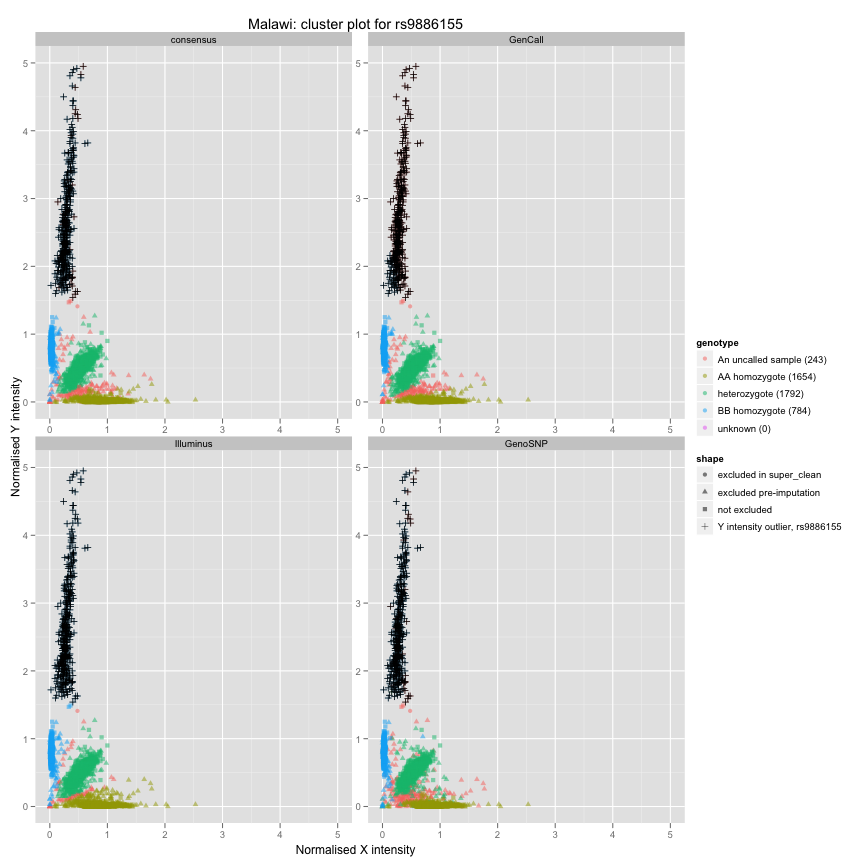

Supplement: Figure S1 — Example of cluster plot from Malawi cohort with outlying sets of individuals. (TIF) [file pgen.1003509.s001.tif]

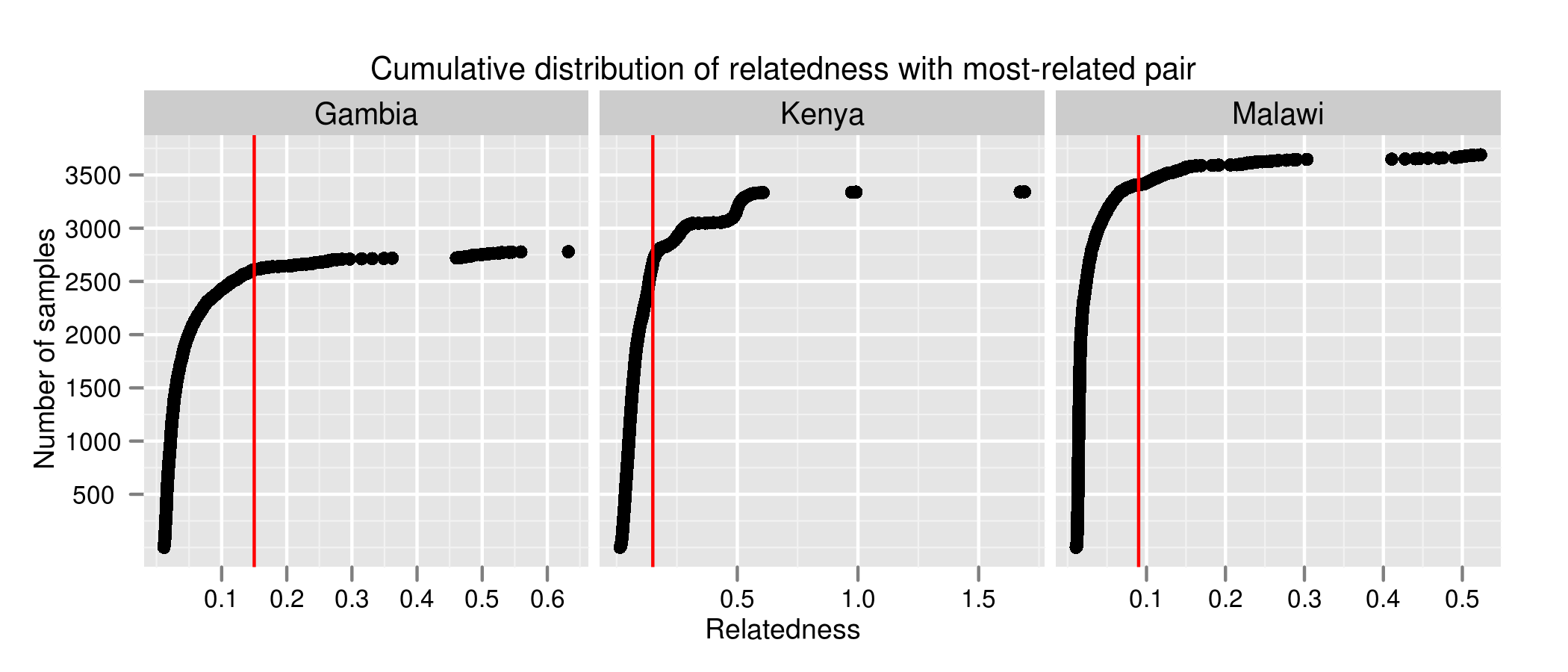

Supplement: Figure S2 — Distribution of relatedness between most-related pairs. (TIF) [file pgen.1003509.s002.tif]

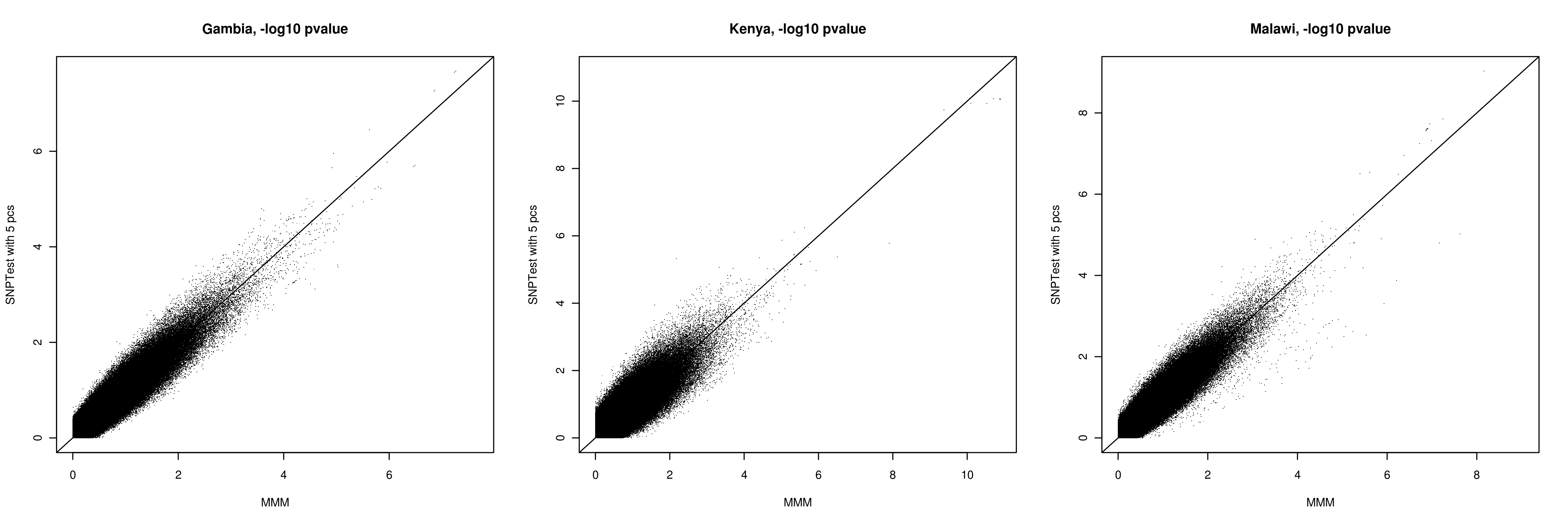

Supplement: Figure S3 — Comparison of logistic regression (SNPTEST) and mixed model (MMM) P values. (TIF) [file pgen.1003509.s003.tif]

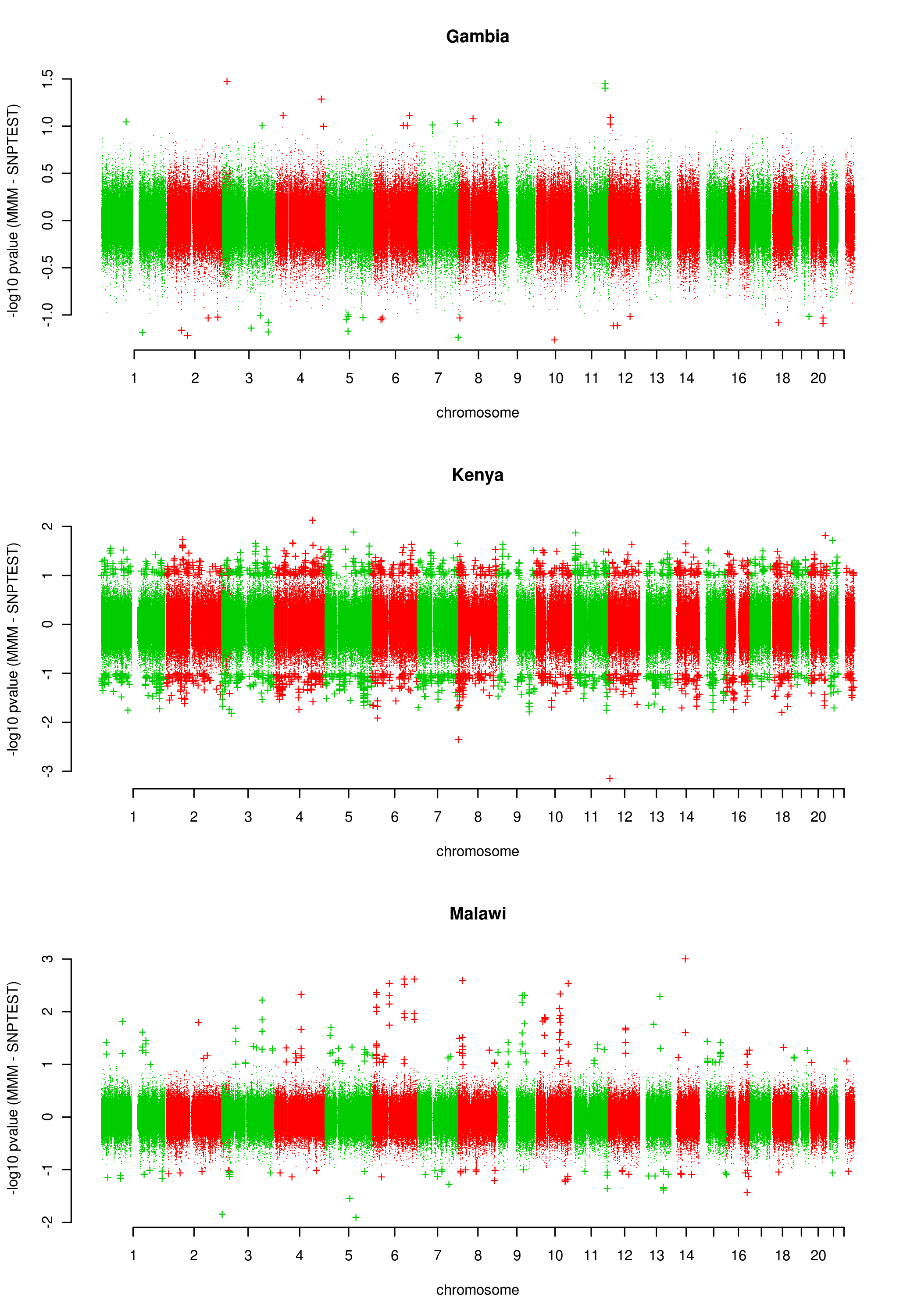

Supplement: Figure S4 — SNPs showing highly divergent P values between logistic regression and mixed model scans. (TIF) [file pgen.1003509.s004.tif]

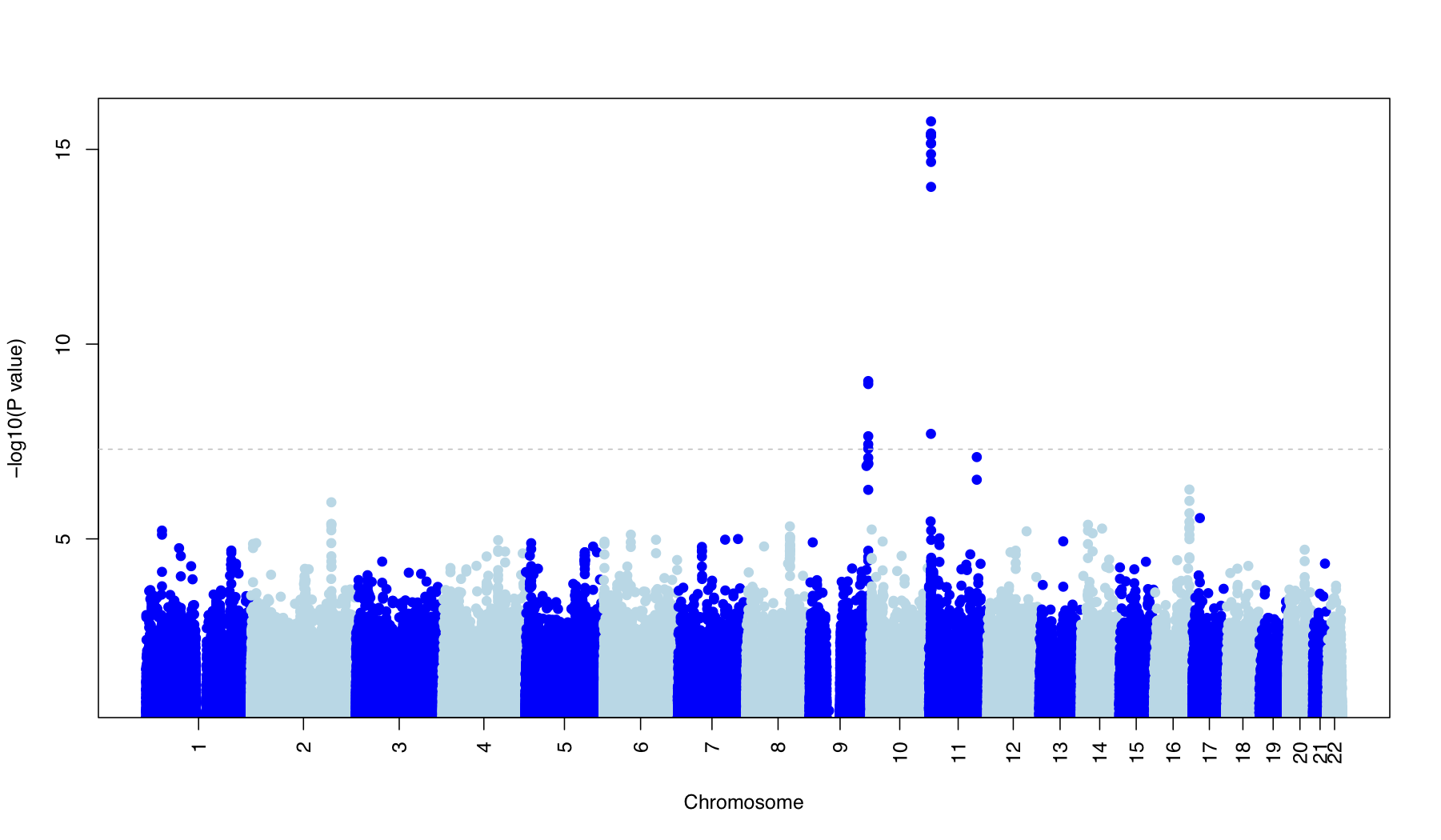

Supplement: Figure S5 — –log10(P values) for test of association using the mixed model. (TIF) [file pgen.1003509.s005.tif]

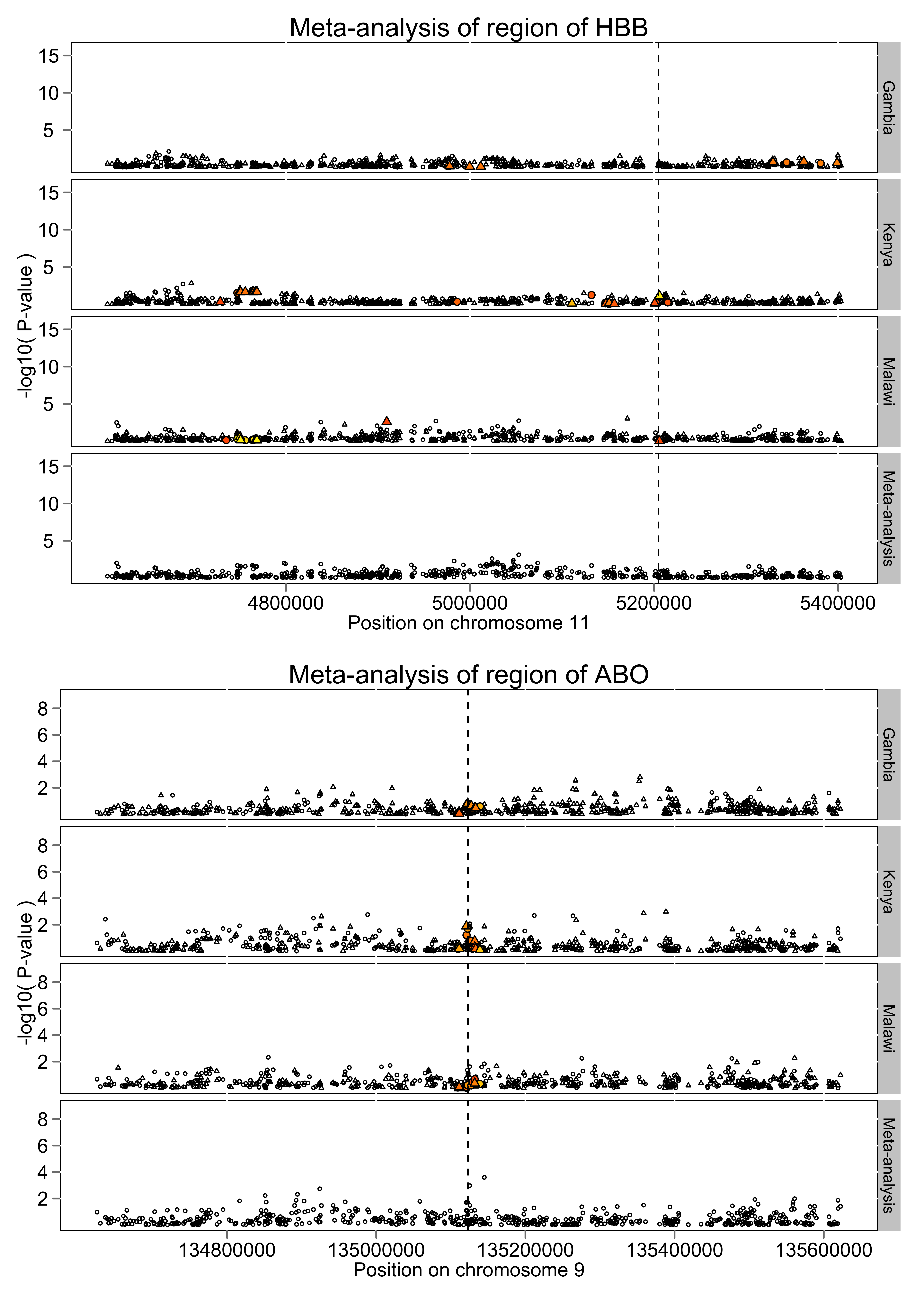

Supplement: Figure S6 — Top: signal of association in the HBB region after conditioning on the genotype at the known causal locus rs334. Bottom: signal of association in the ABO region after conditioning on the genotype at rs8176719. (TIF) [file pgen.1003509.s006.tif]

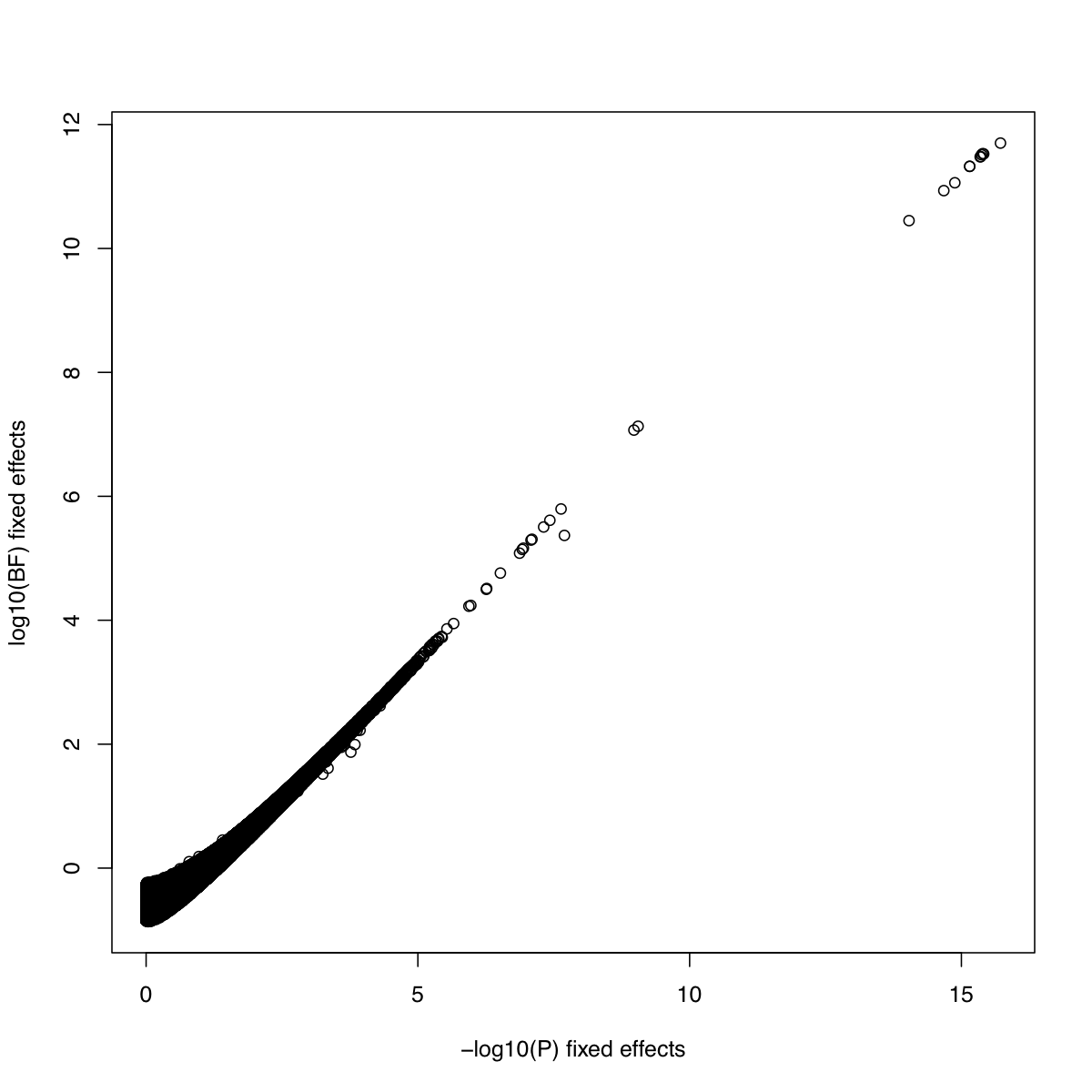

Supplement: Figure S7 — Comparison of meta-analysis P values versus Bayes factors under the fixed-effect model. (TIF) [file pgen.1003509.s007.tif]

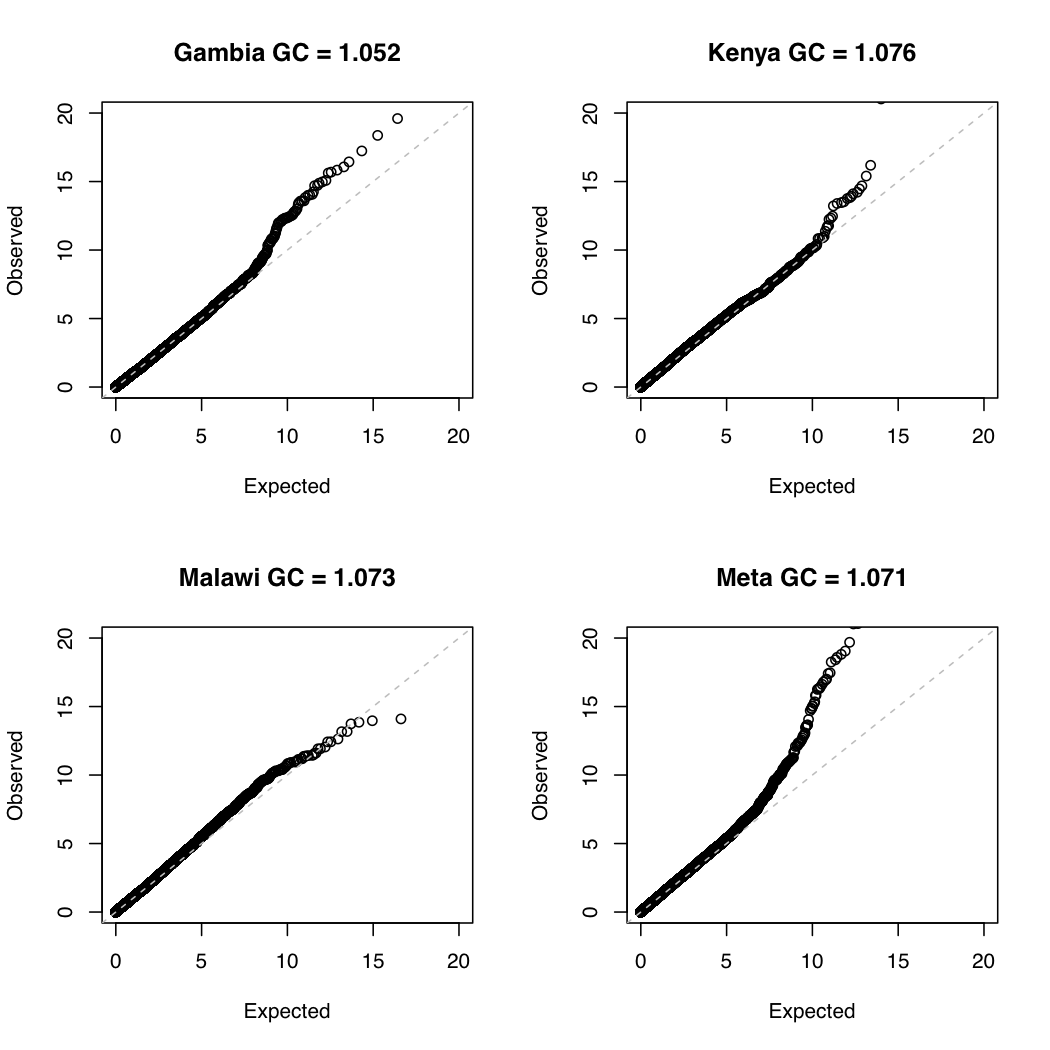

Supplement: Figure S8 — Quantile-quantile plots of the region-based test in the three cohort and in the meta-analysis. The genomic control inflation factor is given in the title of the plots. (TIF) [file pgen.1003509.s008.tif]

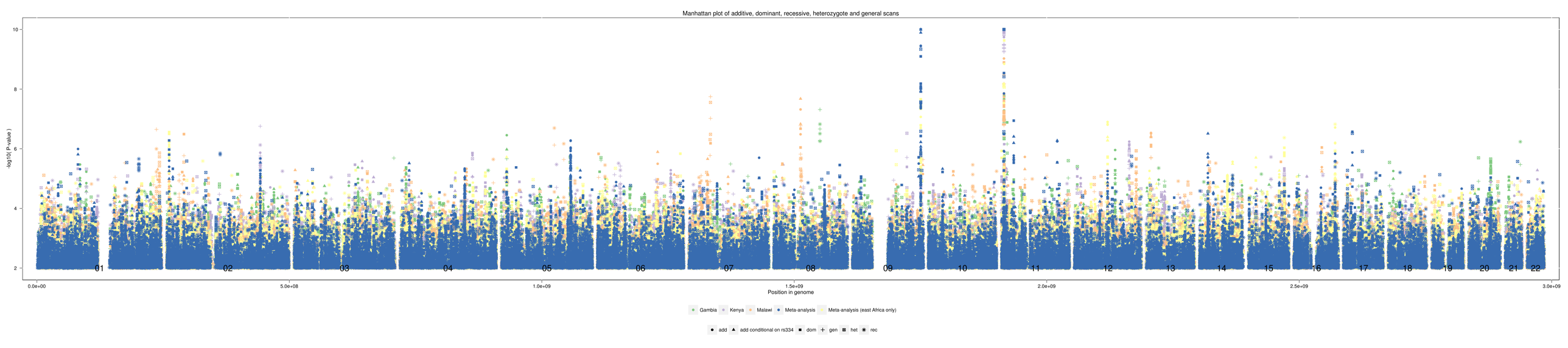

Supplement: Figure S9 — Manhattan plot showing –log10 P values (thresholded at 10) for additive, dominant, heterozygote, recessive, and general models, and additive model conditional on the genotype at the sickle locus rs334, across all imputed SNPs. Meta-analysis P values for all three cohorts and for the East African cohorts are also shown for additive, dominant, recessive and heterozygote scans. (TIF) [file pgen.1003509.s009.tif]

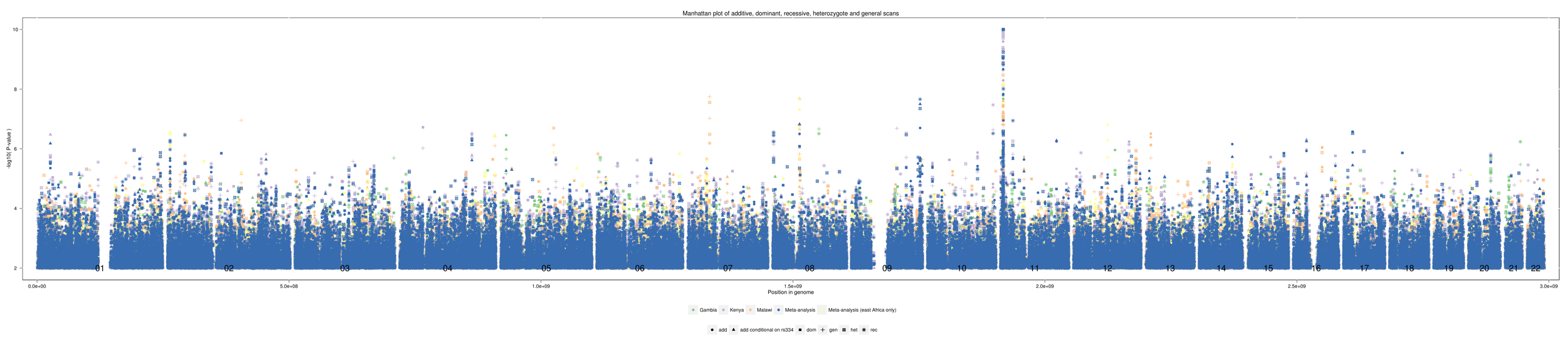

Supplement: Figure S10 — Manhattan plot showing –log10 P values (thresholded at 10) for additive, dominant, heterozygote, recessive, and general models, and additive model conditional on the genotype at the sickle locus rs334, across all non-excluded genotyped SNPs. Meta-analysis P values for all three cohorts and for the East African cohorts are also shown for additive, dominant, recessive and heterozygote scans. (TIF) [file pgen.1003509.s010.tif]

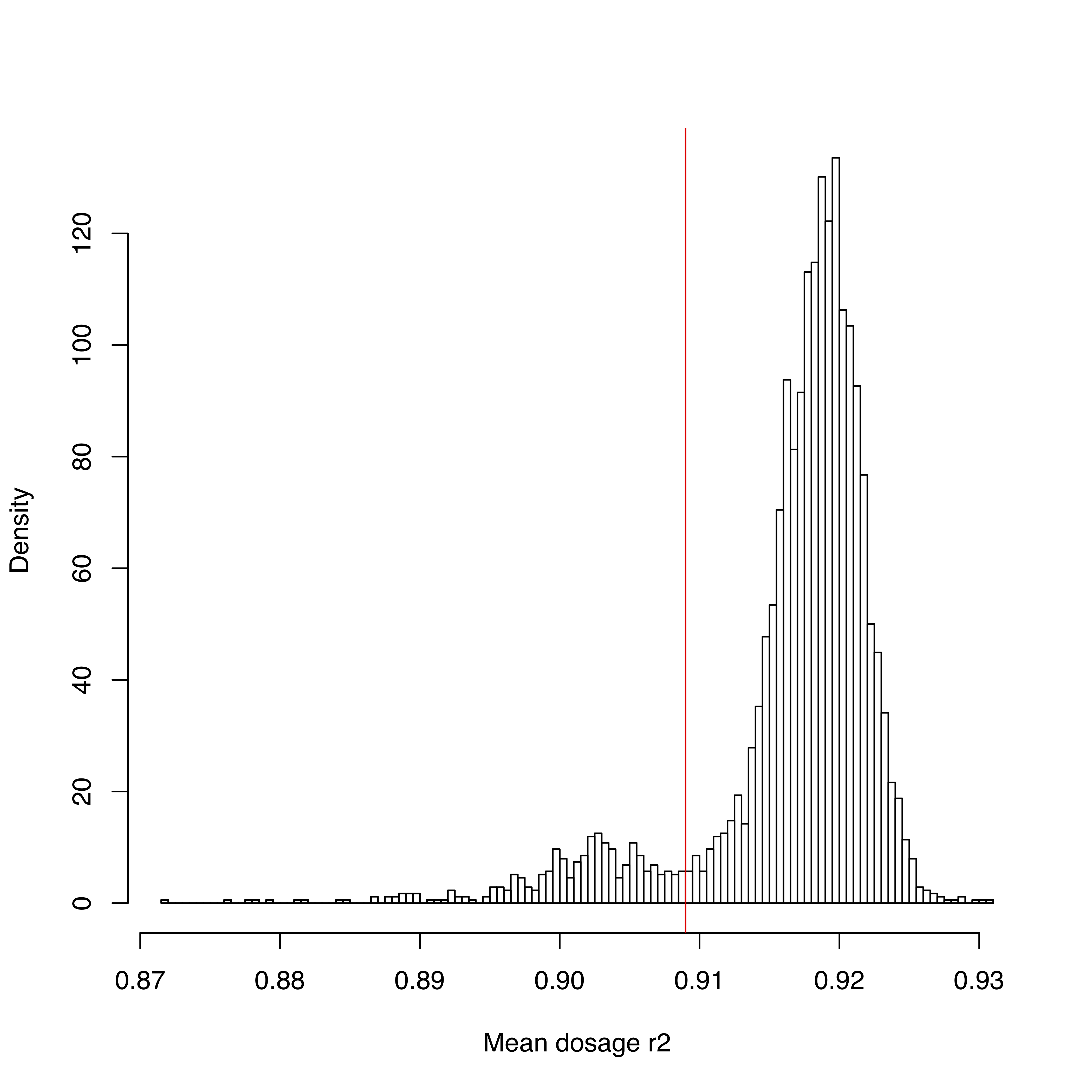

Supplement: Figure S12 — The distribution of imputation quality (measured by type2 r2) across imputed Kenyan samples. The red line is at r2 = 0.909, and is the minimum between the two peaks. (TIF) [file pgen.1003509.s012.tif]

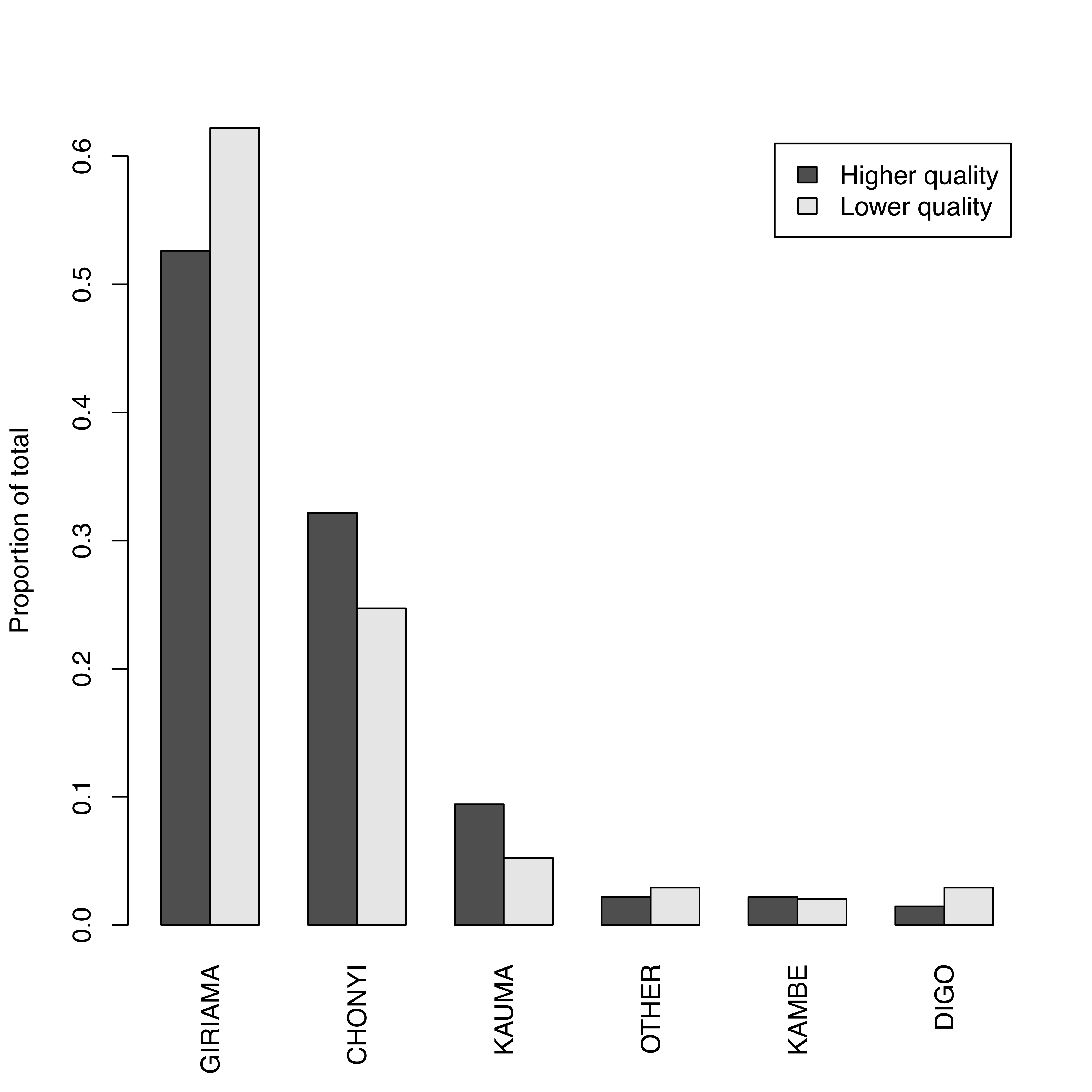

Supplement: Figure S13 — The distribution of ethnic groups in Kenyan samples that were imputed with higher or lower quality (as defined by the red line in Figure S12). The difference in the two distributions is highly significant (Fisher's exact test, P = 4×10^−4^), suggesting that ethnic differences contribute to the bimodal distribution of imputation quality seen in Figure S12. (TIF) [file pgen.1003509.s013.tif]

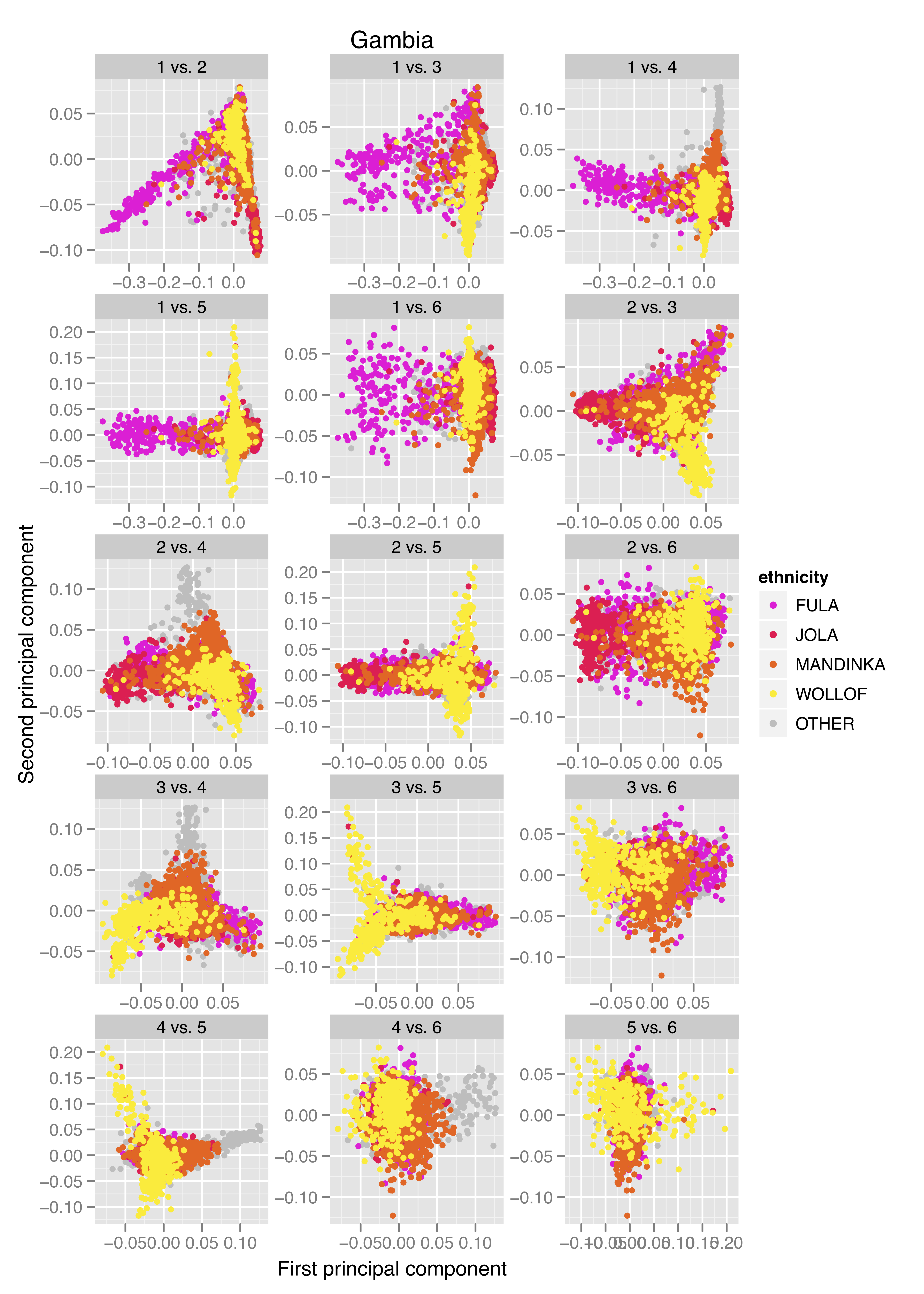

Supplement: Figure S14 — Population-specific PCA analysis of Gambian samples. (TIF) [file pgen.1003509.s014.tif]

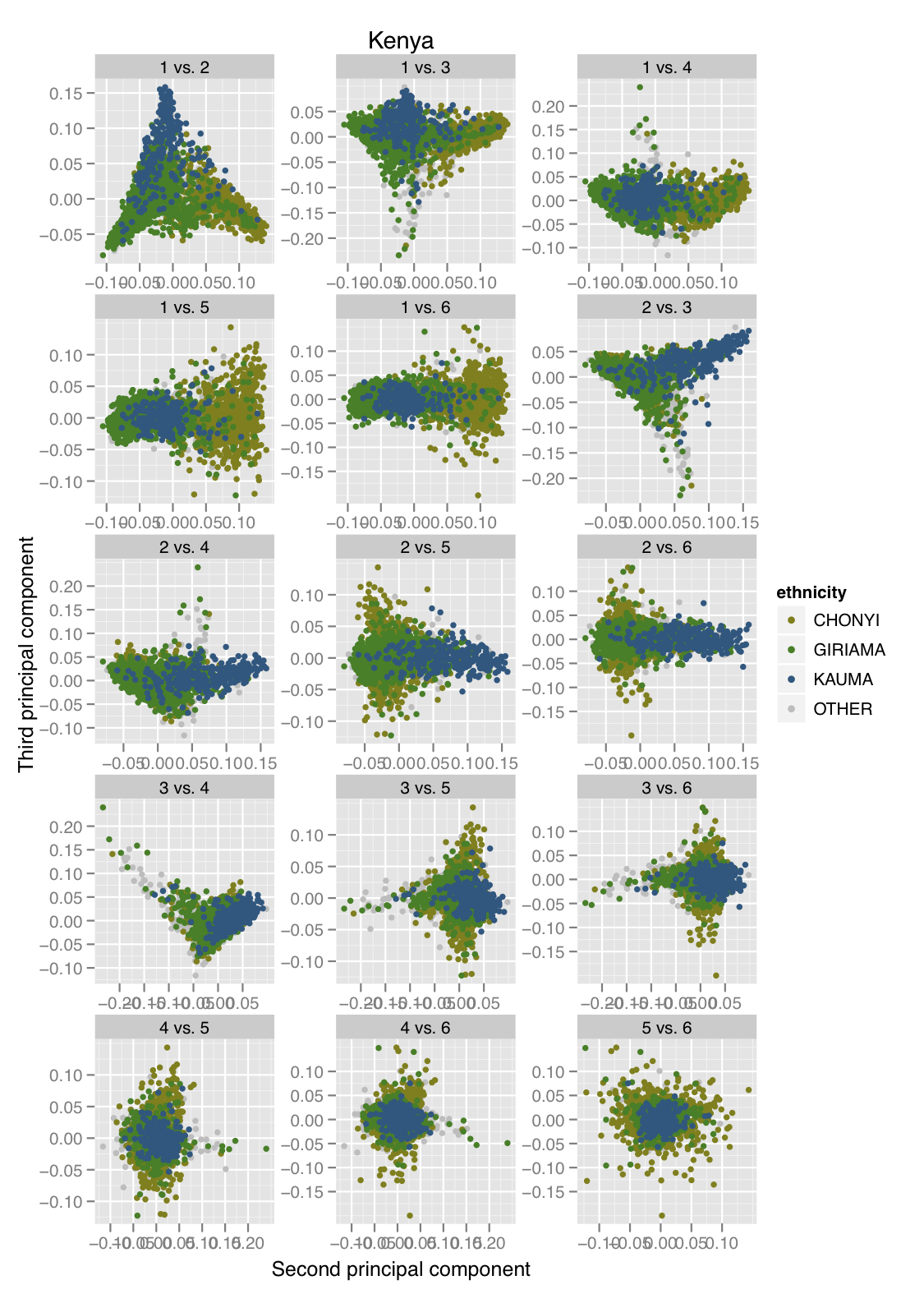

Supplement: Figure S15 — Population-specific PCA analysis of Kenyan samples. (TIF) [file pgen.1003509.s015.tif]

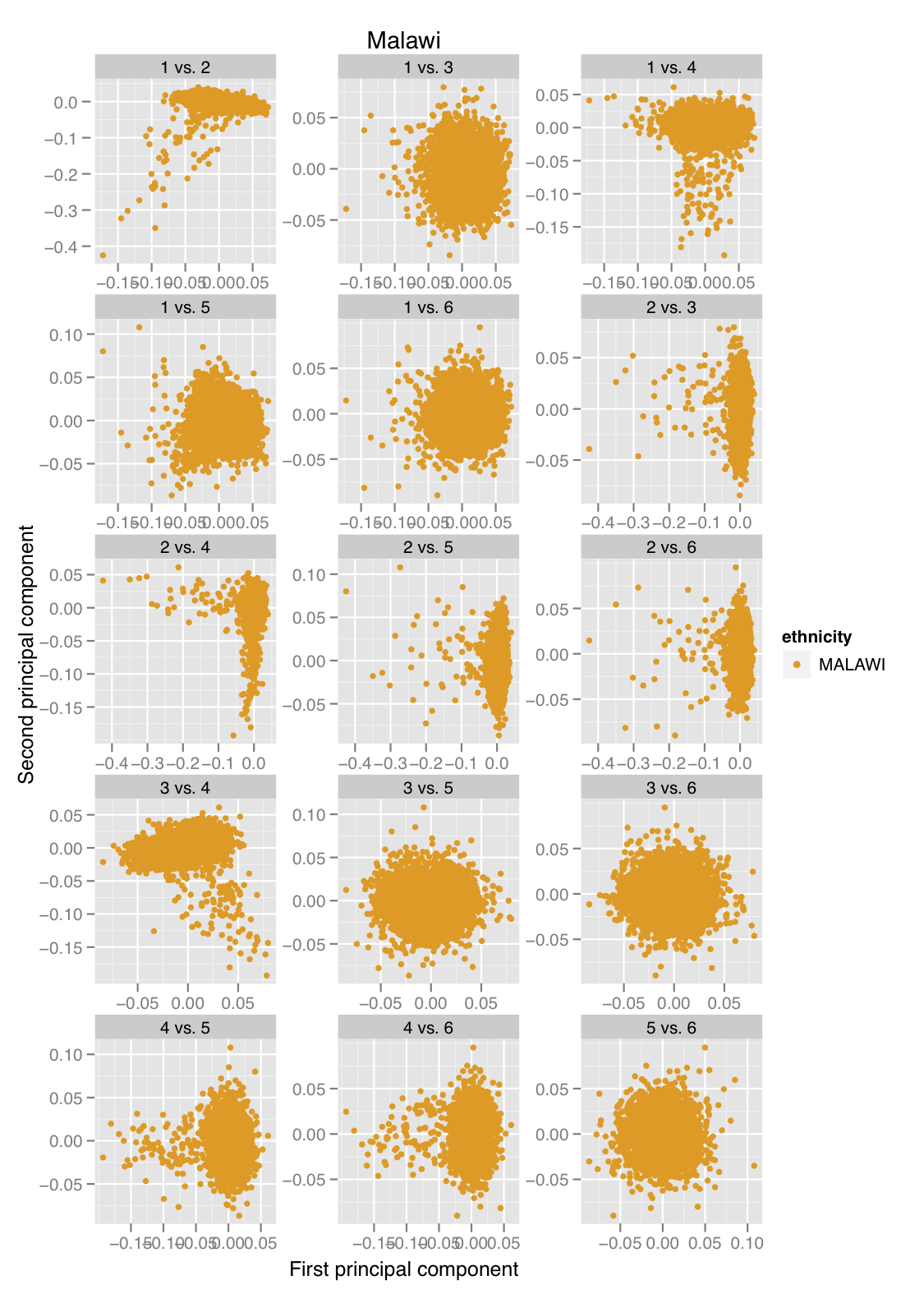

Supplement: Figure S16 — Population-specific PCA analysis of Malawian samples. (TIF) [file pgen.1003509.s016.tif]

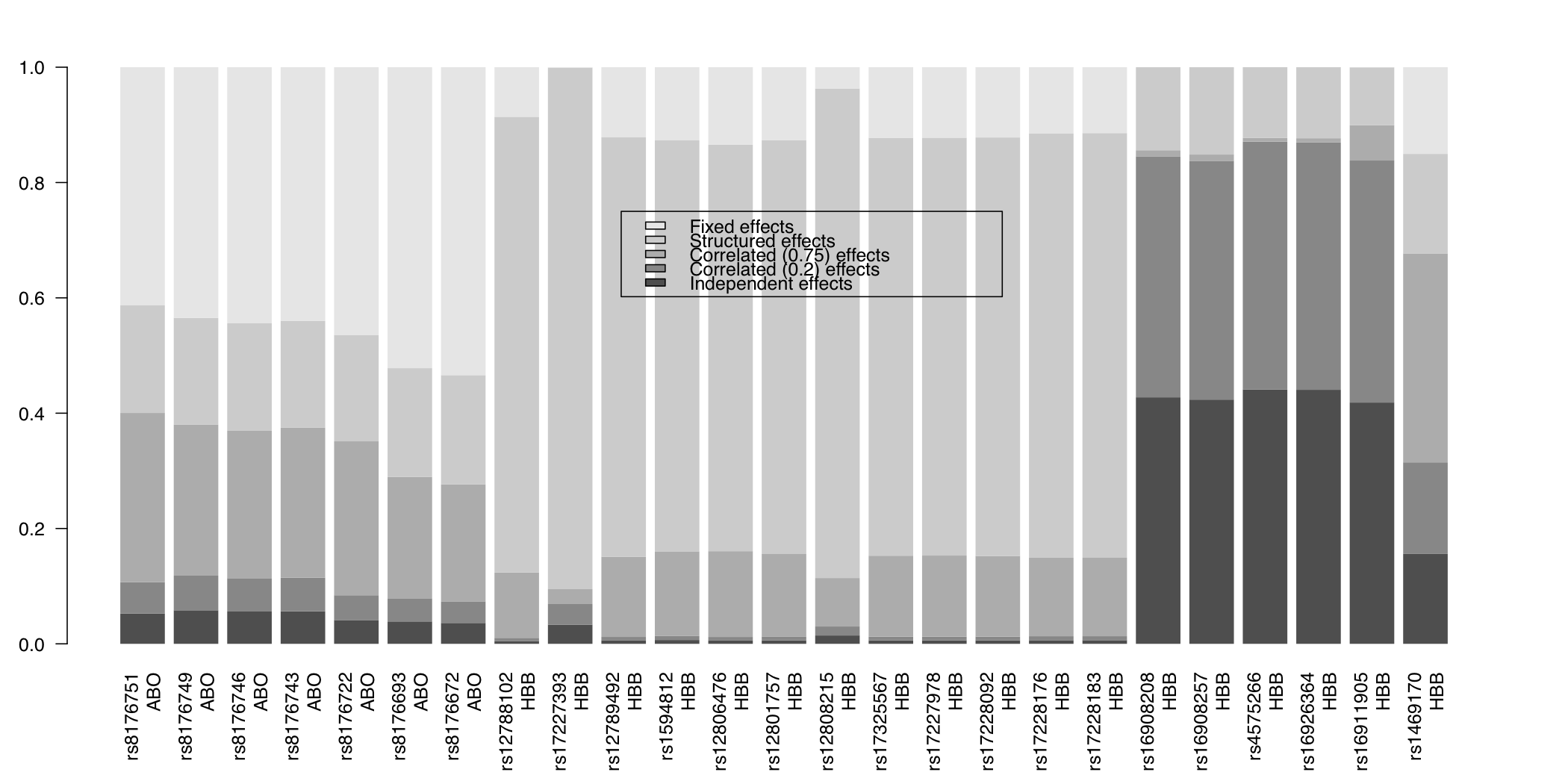

Supplement: Figure S17 — Comparison of fixed, structured, correlated and independent-effect models at the ABO and HBB loci. The height of each bar represents the posterior probability that the corresponding model is true, under the assumption that one of the models is true. (TIF) [file pgen.1003509.s017.tif]

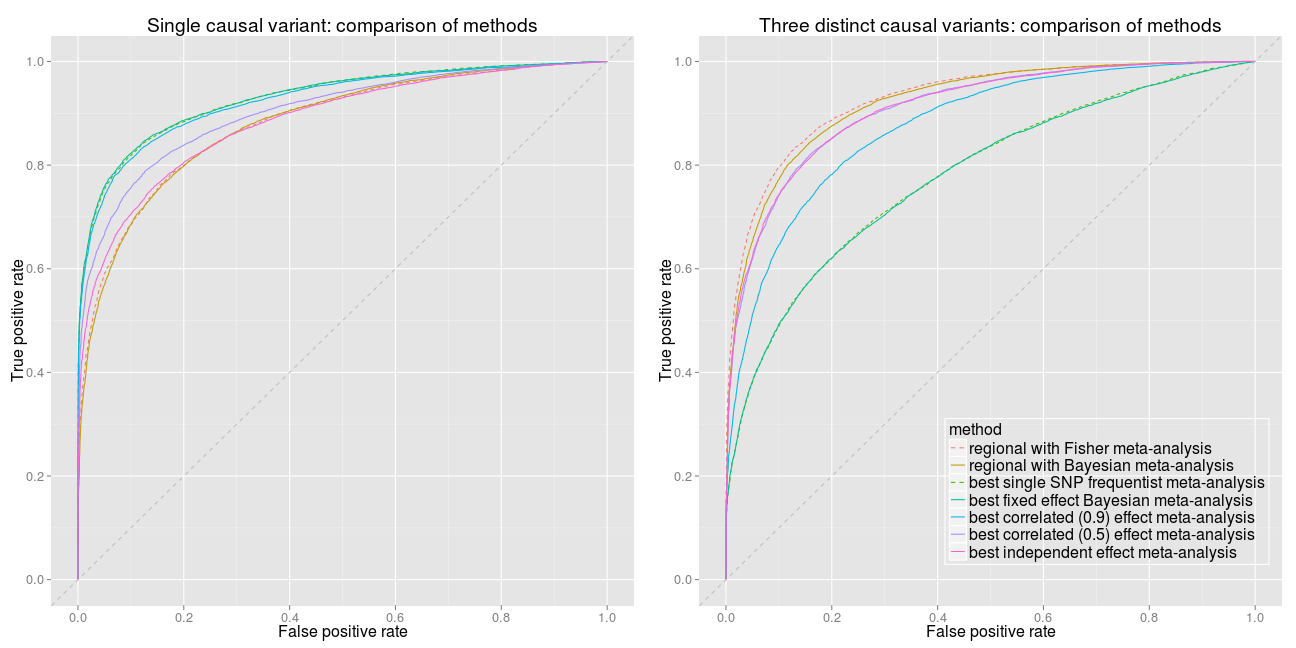

Supplement: Figure S18 — ROC curve showing empirical true positive rate (y-axis) against false positive rate (x-axis) for each method used to detect regional association (regional test with Fisher meta-analysis, regional test with Bayesian meta-analysis, best single-SNP frequentist meta-analysis in region, best single-SNP Bayes factor for each of the four choices of correlation parameter) under the single- variant association scenario (left) and the three-variant association scenario (right). (TIF) [file pgen.1003509.s018.tif]
