# Supplementary material for: Imputation-Based Meta-Analysis of Severe Malaria in Three African Populations
Source: PLoS Genet. 2013 May 23;9(5):e1003509. doi: 10.1371/journal.pgen.1003509 (PMC3662650; doi:10.1371/journal.pgen.1003509)
Supplement: Table S1 — Details on the 3 study sites and genotyping platforms. (DOCX) [file pgen.1003509.s020.docx]

**Supplementary Table S1**: Details on the 3 study sites and genotyping platforms.

| Study | Site | Ethics approving Institution | Ethics Committee | Genotyping Platform |
| --- | --- | --- | --- | --- |
| Gambia | MRC Laboratories, Fajara | MRC Gambia | MRC Ethics Committee | Affymetrix 500K |
| Kenya | KEMRI-Wellcome Research Programme, Kilifi | KEMRI, Kilifi | KEMRI REC | Illumina 2.5 Omni-Quad |
| Malawi | Malawi-Liverpool Wellcome Clinical Research Programme, Blantyre | University of Malawi, college of Medicine | CoM REC | Illumina 1.2M |
