# Supplementary material for: Imputation-Based Meta-Analysis of Severe Malaria in Three African Populations
Source: PLoS Genet. 2013 May 23;9(5):e1003509. doi: 10.1371/journal.pgen.1003509 (PMC3662650; doi:10.1371/journal.pgen.1003509)
Supplement: Table S2 — Pre-imputation individual QC. (DOCX) [file pgen.1003509.s021.docx]

**Supplementary Table S2.** Pre-imputation individual QC.

| Cohort | Total genotyped | Excluded by missingness or heterozygosity | Excluded as duplicate | Total excluded |
| --- | --- | --- | --- | --- |
| Gambia | 4179 | 617 | 782 | 1399 |
| Kenya | 3656 | 134 | 220 | 314 |
| Malawi | 4473 | 594 | 354 | 948 |
