# Supplementary material for: Imputation-Based Meta-Analysis of Severe Malaria in Three African Populations
Source: PLoS Genet. 2013 May 23;9(5):e1003509. doi: 10.1371/journal.pgen.1003509 (PMC3662650; doi:10.1371/journal.pgen.1003509)
Supplement: Table S3 — Pre-imputation SNP QC. (DOCX) [file pgen.1003509.s022.docx]

**Supplementary Table S3.** Pre-imputation SNP QC.

| Cohort | Total #SNPs (total in HapMap III) | Missingness > 2.5% | MAF < 1% | HWE P < 1E-50 | Excluded by Malawi controls | Total excluded |
| --- | --- | --- | --- | --- | --- | --- |
| Gambia | 616255 (569659) | 109981(19%) | 24891(4%) | 2452(<1%) | - | 131887(23%) |
| Kenya | 2450000 (692123) | 54719(7.9%) | 35736(5%) | 473(<1%) | - | 88520(13%) |
| Malawi | 1192668 (909047) | 43737(5%) | 89236(10%) | 1268(<1%) | 2434 | 127713 (14%) |
