# Supplementary material for: Imputation-Based Meta-Analysis of Severe Malaria in Three African Populations
Source: PLoS Genet. 2013 May 23;9(5):e1003509. doi: 10.1371/journal.pgen.1003509 (PMC3662650; doi:10.1371/journal.pgen.1003509)
Supplement: Table S4 — P values for correlation between the first 5 PCs and case/control status. (DOCX) [file pgen.1003509.s023.docx]

**Supplementary Table S4**. *P* values for correlation between the first 5 PCs and case/control status.

|  | PC 1 | PC 2 | PC3 | PC 4 | PC 5 |
| --- | --- | --- | --- | --- | --- |
| Gambia | 1.35e-08 | 7.80e-05 | 0.00742 | 0.03446 | 6.44e-08 |
| Malawi | 1.37e-05 | 0.037366 | 0.047264 | 0.000541 | 0.846552 |
| Kenya | < 2e-16 | 0.16672 | 3.72e-08 | 0.31626 | 0.00596 |
