# Supplementary material for: Imputation-Based Meta-Analysis of Severe Malaria in Three African Populations
Source: PLoS Genet. 2013 May 23;9(5):e1003509. doi: 10.1371/journal.pgen.1003509 (PMC3662650; doi:10.1371/journal.pgen.1003509)
Supplement: Table S5 — Post-imputation sample exclusions. (DOCX) [file pgen.1003509.s024.docx]

**Supplementary Table S5.** Post-imputation sample exclusions.

| Post-imputation sample QC | | |
| --- | --- | --- |
| Cohort | Excluded by relatedness | Excluded as PCA outlier |
| Gambia | 85 | 2 |
| Kenya | 362 | 1 |
| Malawi | 142 | 0 |
