# Supplementary material for: Imputation-Based Meta-Analysis of Severe Malaria in Three African Populations
Source: PLoS Genet. 2013 May 23;9(5):e1003509. doi: 10.1371/journal.pgen.1003509 (PMC3662650; doi:10.1371/journal.pgen.1003509)
Supplement: Table S6 — Genomic Inflation factors (λ) for logistic regression and mixed-model scans. (DOCX) [file pgen.1003509.s025.docx]

**Supplementary Table S6.** Genomic Inflation factors (λ) for logistic regression and mixed-model scans.

|  | SNPTEST (autosomal SNPs) | MMM (autosomal SNPs) |
| --- | --- | --- |
| Gambia | 1.021 | 1.013 |
| Kenya | 1.028 | 1.011 |
| Malawi | 1.016 | 0.999 |
| Meta-analysis | 1.012 | 1.014 |
