# Supplementary material for: Imputation-Based Meta-Analysis of Severe Malaria in Three African Populations
Source: PLoS Genet. 2013 May 23;9(5):e1003509. doi: 10.1371/journal.pgen.1003509 (PMC3662650; doi:10.1371/journal.pgen.1003509)
Supplement: Table S8 — Enrichment of low region based test P values in three previously defined sets of regions. Each P value in the table results from a one-sided binomial test for an enrichment in the number of regions with empirical P value below the given threshold. The bottom row gives a summary of the distribution of the number of SNPs in each region. Note that the Immunochip regions contain on average more SNPs than the gene-based analysis (median = 66 (quartiles = 52, 92)) from which the empirical P value is calculated. (DOCX) [file pgen.1003509.s027.docx]

**Supplementary Table S8.** Enrichment of low region based test *P* values in three previously defined sets of regions. Each *P* value in the table results from a one-sided binomial test for an enrichment in the number of regions with empirical *P* value below the given threshold. The bottom row gives a summary of the distribution of the number of SNPs in each region. Note that the Immunochip regions contain on average more SNPs than the gene-based analysis (median =66 (quartiles = 52, 92)) from which the empirical *P* value is calculated.

| Empirical *P* value threshold | Immunochip regions (n=183) | Haemocyte trait loci (n=96) | Blood group genes (n=26) |
| --- | --- | --- | --- |
| 50% | 0.384 | 0.305 | 0.721 |
| 25% | 0.511 | 0.631 | 0.815 |
| 10% | 0.103 | 0.755 | 0.489 |
| 5% | 0.005 | 0.864 | 0.139 |
| 2.5% | 0.090 | 0.912 | 0.137 |
| 1% | 0.277 | 0.619 | 0.230 |
| SNPs in region  median (quartile) | 98 (50,165) | 66 (36,136) | 52 (23,69) |
