# Supplementary material for: Imputation-Based Meta-Analysis of Severe Malaria in Three African Populations
Source: PLoS Genet. 2013 May 23;9(5):e1003509. doi: 10.1371/journal.pgen.1003509 (PMC3662650; doi:10.1371/journal.pgen.1003509)
Supplement: Text S1 — Details of quality control. (DOCX) [file pgen.1003509.s028.docx]

**Supplementary Text S1:** Details of quality control.

*Sporadic errors in Malawi cohort*

We found that a group of 445 control samples in the Malawi cohort (of which 111 were already excluded by the above criteria) were poorly genotyped at a small number of SNPs spread across the genome. An example is shown in Supplementary Figure S1. At these SNPs, normalized intensities for these samples were very large in either X or Y channel and did not cluster well with the main set of genotypes. However, these samples did not have outlying intensities on average and clustered well at most SNPs. To avoid excluding these samples outright, we performed an association test using these 445 control samples as cases and the remaining other control samples as controls, and excluded any SNP with a *P* value < 1E-04 (Supplementary Table S3). We also examined cluster plots for SNPs that had low *P* values in a standard case/control scan of the Malawi cohort, and removed those SNPs that showed genotype calling problems for these samples.

*Quality control of imputation*

Quality control of the imputation data was undertaken by inspecting plots of measures of genotype accuracy and certainty. Examples of these plots (taken from the imputation of the Kenya dataset) are shown in Supplementary Figure 11. This imputation run has completed without problems, as the quality scores peaks near to 1 (Supp. Figures 11a and 11b), no chunks have abnormally low quality (Supp. Figure 11d), and the imputation performance shows no significant variation genome-wide (Supp. Figure 11e). One anomaly is the unusual “bump” in the per-sample imputation plot (Supp. Figure 11c), which appears to be due to ethnic differences in samples (Supp. Figures 12 and 13).
