# Supplementary material for: Imputation-Based Meta-Analysis of Severe Malaria in Three African Populations
Source: PLoS Genet. 2013 May 23;9(5):e1003509. doi: 10.1371/journal.pgen.1003509 (PMC3662650; doi:10.1371/journal.pgen.1003509)
Supplement: Text S2 — Supplementary statistical details. (PDF) [file pgen.1003509.s029.pdf]

Supplementary statistical details

Chris Spencer, Gavin Band and Matti Pirinen

Approximate Bayes Factors

To allow for possible differences in genetic effects across ethnicities or populations we took a Bayesian approach. Model comparison in the Bayesian framework requires computation of marginal likelihood of the observed data for each of the compared models. For a model M, this means computing the integral

$$\int p(Y|\theta)p(\theta|M)\,d\theta, \tag{1}$$

where Y is the observed data, θ is the vector of the model parameters, $p(Y|\theta)$ is the likelihood function for the parameters and $p(\theta|M)$ is the prior distribution of the parameters under the model M. We use a logistic regression likelihood and treat the case-control status as the data Y. The parameters θ include the intercept, coefficients of any covariates and the allelic SNP effect β, all measured on the log-odds scale.

To simplify the computations, we follow the approach of Wakefield [9, 10]. Within each case-control collection, we approximate the logistic regression likelihood (up to a multiplicative constant) by a multivariate normal density function $f(\theta; \hat{\theta}, \hat{V}_\theta)$, centered at the logistic regression maximum likelihood (ML) estimate $\hat{\theta}$, and having the covariance matrix $\hat{V}_\theta$ which is the inverse of the observed information matrix at $\hat{\theta}$. We use flat priors for other parameters than β. Then the approximate Bayes factor (ABF) for association in the single study reduces to a ratio of marginal likelihood involving only β:

$$\frac{\int f(\beta; \hat{\beta}, SE_\beta^2)p(\beta|M_1)\,d\beta}{\int f(\beta; \hat{\beta}, SE_\beta^2)p(\beta|M_0)\,d\beta}, \tag{2}$$

where SE_β is the estimated standard error of the ML-estimate $\hat{\beta}$, that is, the square root of the diagonal element of the matrix $\hat{V}_\theta$ corresponding to the parameter β.

Under the null model, M_0, we place all our prior probability on $\beta = 0$ and under the alternative we specify $\beta|M_1 \sim N(0, \sigma^2)$. It follows that for any single study the ABF is the ratio of normal densities:

$$ABF = \frac{f(\hat{\beta}; 0, SE_\beta^2 + \sigma^2)}{f(\hat{\beta}; 0, SE_\beta^2)}. \tag{3}$$

This formula leads to a useful interpretation of the approximations made: it is as if we treated the ML point estimate $\hat{\beta}$ and its asymptotic standard error SE_β as observed data which define a normal likelihood function over the parameters of interest. In practice, $\hat{\beta}$ and SE_β can be obtained from ML approaches implemented for logistic regression in standard statistical software packages such as R

To extend the result to a meta-analysis of (say) three independent studies, we will replace the joint likelihood function of all the parameters by the density $f(\beta; \hat{\beta}, \hat{V_\beta})$ where $\beta = (\beta_1, \beta_2, \beta_3)$ and $\hat{V_\beta} = \text{diag}(SE^2_{\beta_1}, SE^2_{\beta_2}, SE^2_{\beta_3})$.

Calculation of the marginal likelihood requires specifying a prior distribution for the study-wise effect sizes β_1, β_2 and β_3. We choose a multivariate normal distribution with zero mean. To allow us to specify different prior beliefs about the similarity in effect sizes across populations, we use a covariance matrix of the form

$$\sigma^2 \begin{bmatrix} 1 & \rho_{12} & \rho_{13} \\ \rho_{21} & 1 & \rho_{23} \\ \rho_{31} & \rho_{32} & 1 \end{bmatrix}, \tag{4}$$

where $\rho_{ij} = \rho_{ji}$ is the correlation in effect size between the studies i and j. The approximate marginal likelihood for any given choice of prior parameters is then (up to a multiplicative constant which is independent of the prior parameters) the value of a multivariate normal density at the ML estimate,

$$f\left(\begin{bmatrix} \hat{\beta}_1 \\ \hat{\beta}_2 \\ \hat{\beta}_3 \end{bmatrix}; \begin{bmatrix} 0 \\ 0 \\ 0 \end{bmatrix}, \sigma^2 \begin{bmatrix} 1 & \rho_{12} & \rho_{13} \\ \rho_{21} & 1 & \rho_{23} \\ \rho_{31} & \rho_{32} & 1 \end{bmatrix} + \begin{bmatrix} SE^2_{\beta_1} & 0 & 0 \\ 0 & SE^2_{\beta_2} & 0 \\ 0 & 0 & SE^2_{\beta_3} \end{bmatrix} \right). \tag{5}$$

By choosing different values for the correlation parameters we can compare different models of association to the null model of no association.

1. **Fixed effects model**; $\rho_{12} = \rho_{13} = \rho_{23} = 1$. The standard meta-analysis fixed effects model can be recovered by setting the correlation between the true effect sizes between cohorts to one. It is interesting to note that exactly the same ABF is obtained if the estimated effect sizes are first combined using a frequentist inverse-variance weighted meta-analysis to produce a study-wide $\hat{\beta}$ and SE_β, and these values are substituted in equation 3.

2. **Independent effects model**; $\rho_{12} = \rho_{13} = \rho_{23} = 0$. The independent effects model explicitly assumes that a priori there is no correlation in effect sizes across studies. In this case the marginal likelihood factorises into terms for each study, and the study-wide ABF comparing models of association to no association can be obtained by multiplying the study-wise approximate Bayes factors as given in equation (3). Note that this makes

clear that frequentist meta-analysis approaches, which combine either p-values or chi-squared statistics across populations, implicitly assume an independent effects model.

3. **Correlated effects model**; $\rho_{12} = \rho_{13} = \rho_{23}$. In between the two models described above we can specify a prior whereby the effects are similar between models, but importantly, not necessarily the same. We use the correlated effects model to refer to a prior where all pairs of the studies have the same correlation in effect size.

4. **Structured effects model**; The most general form of the model allows arbitrary correlation between effects across studies. This is appropriate if there is a reason to believe *a priori* that some studies may have more similar effects than others.

These scenarios represent four broad categories of assumption on effect size across studies. We note that the models 3 and 4 have similarities with a hierarchical random effects model where the effect of each population is chosen from a common distribution, but we do not discuss these connections further here. Moreover, we do not investigate models where an unknown subset of the studies has no effect ($\beta = 0$).

Evidence for heterogeneity through model comparison

As well as computing ABFs for comparing models of association to the null model of no association, we can also compare different models of association. For example, under the assumption that exactly one of the above four models is correct, (so the space of possible models is (M_1, M_2, M_3, M_4) as enumerated above), we can calculate the posterior probability of the model M_i as

$$P(M_i|data) = \frac{ABF_{M_i} P(M_i)}{\sum_i (ABF_{M_i} P(M_i))}, \quad (6)$$

where ABF_{M_i} is the approximate Bayes factor comparing model i to the null model of no association, and $P(M_i)$ is the prior probability of model M_i. For example, by computing the ratio of the ABFs for models 1 and 2 we could assess the evidence for heterogeneity of effects, and by specifying a prior on the model space, we could compute the posterior probability of M_1 using equation (6).

Marginal priors on effect sizes

The models above all assume a normal prior distribution on the effect size. If, as is the case in our study, the estimated effects are derived by fitting a logistic regression model which assumes that each copy of the risk allele increase odds of the disease multiplicatively, then e^{β} is the odds ratio (or relative risk). In several Bayesian applications in GWAS, the prior on the effect size on the log odds scale has been centered at zero with standard deviation $\sigma = 0.2$ [11]. In our

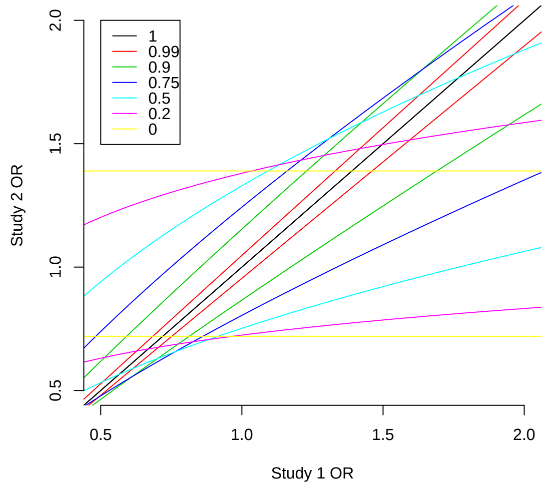

Figure 1: The 95% highest probability regions of the conditional distribution of the odds ratio (OR) in study 2 conditional on the OR in study 1 for different prior assumptions on the correlation in OR between the two studies (given in the legend).

formulation of the meta-analysis approach we assume the same marginal prior on effect size within each study, although it is straight forward to relax this assumption. Here we specify a correlation in the odds ratios between studies, which can be plotted as a conditional distribution. For example Figure 1 shows probability regions on the effect size in a second study (study 2) given the effect size in the first study, for different positive correlations on the log-odds scale.

Region-based test

When the true causal variants are not genotyped directly or when a region contains many tiny effects that are too small be identified individually, a proper region-based test may be more powerful than a traditional single-SNP analysis. For region-based tests we use a software package MMM [8] for inference using a linear mixed model as an approximation to logistic regression. The linear mixed model can be written as

$$Y = \alpha + X\beta + Z + \epsilon. \tag{7}$$

where Y is a binary label of case-control status, α is the baseline effect, and X is a matrix of covariates and predictors with associated linear effects β.

The random effects include the usual error term ϵ, and one additional effect Z parameterised and distributed as follows:

$$Z|(h,\sigma^2) \sim N(0, h\sigma^2 R) \text{ and } \epsilon|(h,\sigma^2) \sim N(0,(1-h)\sigma^2 I), \tag{8}$$

where $h \in [0,1]$ and $\sigma^2 > 0$ are scalars that define how the variance is decomposed between Z and ϵ and R is an $n \times n$ covariance matrix and I is the corresponding identity matrix.

Models of this form have been shown to be useful in GWAS for correcting for relatedness structure by using the additional random effect Z to model the correlation in phenotypes due to patterns of relatedness between study individuals [13, 6, 5, 14, 8]. These approaches compute the matrix R from the correlation in genotypes between the individuals at an approximately uncorrelated set of SNPs across the autosomes. A statistical test is then constructed to ask whether a SNP (a column of X) has an effect on the disease risk after accounting for putatively confounding effects of the relatedness structure R. The model can be efficiently applied genome-wide by an algorithm that requires a single eigenvalue decomposition of R and is implemented in a software package MMM [8]. This linear mixed model approach was also applied in our study to carry out single SNP analyses.

For region-based tests we applied the same linear mixed model but this time by including a small number of leading eigenvectors of the genome-wide correlation matrix as fixed effects (in X), in order to account for population structure, and then determining the relatedness matrix R locally using all the SNPs within a focused region of the genome (and having minor allele frequency $\geq 1\%$). Instead of comparing single-SNP models where an element of β, corresponding to the SNP effect, is zero (null model) or non-zero (alternative model), we tested whether h is non-zero. The test is asking whether the "heritability" (i.e. variance explained by additive genetic effects) attributed to the region is significantly different from zero. Similar test has been recently considered by [7].

As each region of the genome defines its own R matrix, we lose the computational efficiency exploited in the program MMM. However, for a data sets of the size analysed here (up to 5,000 samples per collection) the required matrix decomposition for any region takes less than 10 minutes on a standard processor, and the whole analysis was feasible by using a computing cluster.

Further details

There are at least two ways of conceptualizing a test based on local patterns of relatedness. Perhaps the most helpful is to rewrite the random effect Z as a sum of the contribution of each SNP, which is how the model was recently motivated to estimate heritability [12] and to define a region-based test [7]. Let the vector of genotypes across individuals at SNP i be g_i and let p_i be the corresponding frequency of the allele 1. Consider the model

$$Y = \alpha + X\beta + \sum_{i=1}^{L} g_i^* \gamma_i + \epsilon, \tag{9}$$

where $g_i^* = (g_i - 2p_i)/\sqrt{2p_i(1-p_i)}$ contains the standardised genotypes, L is the total number of SNPs in the region and the random SNP effect is distributed as

$$\gamma_i \sim N\left(0, h\sigma^2/L\right). \tag{10}$$

Here we see that the contribution of each standardised SNP g_i^* is weighted by a random effect γ_i. It can be shown that the random sum in (9) has a covariance matrix given by $h\sigma^2 R$, where R is an empirical genotypic correlation matrix computed over the L SNPs. Thus by standardising the genotypes we make clear the equivalence with the model defined by (7) and (8). It is insightful to replace the standardised g_i^* with the mean centered raw genotypes $g_i - 2p_i$ in equation (9) and note that then equation (10) becomes

$$\gamma_i \sim N\left(0, \frac{h\sigma^2}{2p_i(1-p_i)L}\right). \tag{11}$$

From this it is clear that setting R to a genotypic correlation matrix implies that rarer SNPs are allowed to have larger effect sizes.

Another way to write the model is to consider R, defined by the patterns of allele sharing within a region, in terms of its eigenvalue decomposition $R = UDU^T$, where U is an orthonormal matrix of eigenvectors and D is a diagonal matrix of the corresponding eigenvalues. Then the equation (9) can be written as

$$Y = \alpha + X\beta + \sum_{i=1}^{n} U_i \xi_i + \epsilon, \tag{12}$$

where

$$\xi_i \sim N\left(0, d_i h\sigma^2\right). \tag{13}$$

Here U_i is the ith eigenvector of R (also the vector of positions of each individual on the ith principal component), and d_i is the associated eigenvalue. The models defined by equation (9) and (12) are equivalent because the correlation between the standardised genotypes of two individuals (an element of R) is the same as the inner product of the positions of two individuals in the principal components weighted by the proportion of variance that each PCs explain. Written in this way we see that the test is equivalent to including all principal components of the local relatedness matrix in a Bayesian linear regression with priors that are proportional to the corresponding eigenvalues. (See also [1].)

Testing for association

All the computations were done with the software package MMM [8].

Calculating p -values

When $h = 0$ and R is not a block-diagonal matrix, the distribution of the likelihood-ratio statistic from the linear mixed model (7) does not necessarily follow the standard null distribution of a 50:50 mixture of a point mass at 0 and a χ_1^2 distribution [2]. Empirically we found that this standard distribution of $0.5\delta_0 + 0.5\chi_1^2$ is conservative as the mass at zero was significantly larger than 0.5. Thus the reported p-values from the likelihood ratio test are likely to be conservative.

As another option we used a score statistic for testing $h = 0$:

$$\sum_{i=1}^{n}(d_i - 1)\frac{(\tilde{Y}_i - \tilde{X}_i\hat{\beta})^2}{\widehat{\sigma^2}}, \tag{14}$$

where $\tilde{Y} = U^T Y$ and $\tilde{X} = U^T X$ are transformed data and predictors, U is the matrix of eigenvectors of R and $(d_i)_{i=1}^n$ are the corresponding eigenvalues and the ML-estimates $\hat{\beta}$ and $\widehat{\sigma^2}$ come from the null model where $h = 0$.

Under the null model $h = 0$, the score statistic (14) is distributed as a mixture

$$\sum_{i=1}^{n}(d_i - 1)\chi_{1,i}^2,$$

where each $\chi_{1,i}^2$ is an independent draw from the central chi-square distribution with one degree of freedom. Our software MMM implements the p-value computations from this distribution by using Davies method [3] as recently implemented in the R-package CompQuadForm [4]. Previously, similar approach has been used by [7].

Calculating Bayes factors

The marginal likelihood computations required for the Bayesian model comparison have been implemented in the software package MMM. The null model is $h = 0$ and for the alternative model we have used the prior $h \sim \text{Beta}(1.5, 100)$ having a mean of 0.0148. We focused the alternative model to relatively small values of h as we expect that any tested region explains at most a small proportion of the total variance. For the other parameters (β and σ^2) we used the same Normal-inverse-gamma prior in both models.

References

[1] W Astle and DJ Balding. Population structure and cryptic relatedness in genetic association studies. *Stat Sci*, 24:451–471, 2009.

[2] CM Crainiceanu and D Ruppert. Likelihood ratio tests in linear mixed models with one variance component. *J R Statist Soc B*, 66:165–185, 2004.

[3] RB Davies. Algorithm as 155: The distribution of a linear combination of χ^2 squared random variables. *J R Statist Soc C*, 29:323–333, 1980.

[4] P. Duchesne and P. Lafaye de Micheaux. Computing the distribution of quadratic forms: Further comparisons between the liu-tang-zhang approximation and exactmethods. *Computational Statistics and Data Analysis*, 54:858–862, 2010.

[5] HM Kang, JH Sul, SK Service, NA Zaitlen, S Kong, NB Freimer, C Sabatti, and E Eskin. Variance component model to account for sample structure in genome-wide association studies. *Nat Genet*, 42:348–354, 2010.

[6] HM Kang, NA Zaitlen, CM Wade, A Kirby, D Heckerman, MJ Daly, and E Eskin. Efficient control of population structure in model organism association mapping. *Genetics*, 178:1709–1723, 2008.

[7] T Cai Y Li M Boehnke X Lin MC Wu, S Lee. Rare-variant association testing for sequencing data with the sequence kernel association test. *Am J Hum Genet*, 89:82–93, 2011.

[8] M Pirinen, P Donnelly, and C Spencer. Efficient computation with a linear mixed model on large-scale data sets with applications to genetic studies. *Ann Appl Stat*, in press, 2012.

[9] J. Wakefield. A Bayesian measure of the probability of false discovery in genetic epidemiology studies. *Am. J. Hum. Genet.*, 81(2):208–227, Aug 2007.

[10] J Wakefield. Bayes factors for genome-wide association studies: comparison with p-values. *Gen Epidem*, 33:79–86, 2009.

[11] WTCCC. Genome-wide association study of 14,000 cases of seven common diseases and 3,000 shared controls. *Nature*, 447:661–678, 2007.

[12] J Yang, B Benyamin, BP McEvoy, S Gordon, AK Henders, DR Nyholt, PA Madden, AC Heath, NG Martin, GW Montgomery, ME Goddard, and PM Visscher. Common snps explain a large proportion of the heritability for human height. *Nat Gen*, 42:565–569, 2010.

[13] J Yu, G Pressoir, WH Briggs, IV Bi, M Yamasaki, JF Doebley, MD McMullen, BS Gaut, DM Nielsen, JB Holland, S Kresovich, and ES Buckler. A unified mixed-model method for association mapping that accounts for multiple levels of relatedness. *Nat Gen*, 38:203–208, 2005.

[14] Z Zhang, E Ersoz, CQ Lai, RJ Todhunter, HK Tiwari, MA Gore, JM Bradbury, J Yu, DK Arnett, JM Ordovas, and ES Buckler. Mixed linear model approach adapted for genome-wide association studies. *Nat Gen*, 42:355–360, 2010.
